# A SARS-CoV-2 NSP7 homolog of a Treg epitope suppresses CD4+ and CD8+ T cell memory responses

**DOI:** 10.3389/fimmu.2023.1290688

**Published:** 2023-11-24

**Authors:** S. M. Shahjahan Miah, Sandra Lelias, Andres H. Gutierrez, Mitchell McAllister, Christine M. Boyle, Lenny Moise, Anne S. De Groot

**Affiliations:** ^1^ EpiVax, Inc., Providence, RI, United States; ^2^ Center for Vaccines and Immunology, University of Georgia, Athens, GA, United States

**Keywords:** SARS-CoV-2, Tregitope, nsp7, tolerance, immunogenicity, immune camouflage

## Abstract

Pathogens escape host defenses by T-cell epitope mutation or deletion (immune escape) and by simulating the appearance of human T cell epitopes (immune camouflage). We identified a highly conserved, human-like T cell epitope in non-structural protein 7 (NSP7) of SARS-CoV-2, RNA-dependent RNA polymerase (RdRp) hetero-tetramer complex. Remarkably, this T cell epitope has significant homology to a T regulatory cell epitope (Tregitope) previously identified in the Fc region of human immunoglobulin G (IgG) (Tregitope 289). We hypothesized that the SARS-CoV-2 NSP7 epitope (NSP7-289) may induce suppressive responses by engaging and activating pre-existing regulatory T cells. We therefore compared NSP7-289 and IgG Tregitopes (289 and 289z, a shorter version of 289 that isolates the shared NSP7 epitope) *in vitro*. Tregitope peptides 289, 289z and NSP7-289 bound to multiple HLA-DRB1 alleles *in vitro* and suppressed CD4+ and CD8+ T cell memory responses. Identification and *in vitro* validation of SARS-CoV-2 NSP7-289 provides further evidence of immune camouflage and suggests that pathogens can use human-like epitopes to evade immune response and potentially enhance host tolerance. Further exploration of the role of cross-conserved Tregs in human immune responses to pathogens such as SARS-CoV-2 is warranted.

## 1 Introduction

The COVID-19 pandemic caused by SARS-CoV-2 (severe acute respiratory syndrome coronavirus 2) resulted in unprecedented loss of life and a worldwide economic crisis. The global impact of the pandemic is highlighted by 689 million cases and 6.8 million deaths which is likely to be underestimated (1). Vaccine and natural infection studies of SARS-CoV-2 show that both antibody and T cell responses are critical to obtaining protective immunity (2, 3). To better understand the T cell response to SARS-CoV-2, we screened for potential regulatory T cell epitopes (Tregitopes). Here, we report the discovery of a Tregitope in non-structural protein 7 (NSP7-289) of SARS-CoV-2.

Not all T cell epitopes that are found in human pathogens are ‘effector’, or inflammatory T cell epitopes. Some pathogens contain Tregitopes that activate regulatory T cell responses in their human hosts, which can suppress the host immune response (4). Key features of Tregitopes are that they are HLA-DR-binding T cell epitopes with T cell receptor (TCR)-facing amino acid residues that are highly cross-conserved with immunoglobulin and other human protein sequences. In 2013, we discovered that many human pathogens contained Tregitopes (5, 6). The presences of Tregitopes may allow viruses to reduce the potential for an immune response to the viral antigen (7). This concept of dissimulation by Treg known as immune camouflage (5–9), is similar to the concept known as epitope mimicry that has been reported previously (10, 11). Immune camouflage refers to the presence of human-like tolerizing epitopes that may be integrated into key antigens by pathogens (over the course of pathogen evolution with human hosts) that enable the pathogens to escape immune attack. Immune camouflage may help viruses avoid natural immune defenses, and in the case where the viral epitope is recognized by a Treg it may also lead to suppression of immune response (12).

SARS-CoV-2 NSP7-289 is highly homologous (at T cell receptor-facing residues) with a well-defined and validated human immunoglobulin G (IgG) Tregitope, known as Tregitope 289. De Groot et al. in 2008 (13) identified IgG Tregitopes (Tregitope 289 and 167) as highly conserved natural regulatory T cell (nTregs) epitopes that engage and activate nTregs. The role of IgG Tregitopes may be to reduce anti-idiotypic immune response, as V(D)J recombination and somatic hypermutation lead to the development of complementary-determining regions (CDRs) that appear foreign to self.

Since NSP7 is a component of the RNA-dependent RNA polymerase (RdRp) that is essential for viral replication and survival, we hypothesize that NSP7-289 may modulate immune response to the viral-encoded RNA replicase and contribute to T cell suppression (7). In this study, we investigated NSP7-289 binding to HLA-DRB1 supertype alleles and its ability to suppress effector T cells *in vitro*.

## 2 Materials and methods

### 2.1 NSP7 sequences

SARS-CoV-2 (taxid: 2697049), SARS-CoV (taxid: 694009), MERS-CoV (taxid: 1335626), and human CoV (taxids: 11137, 443239, 277944, and 31631) NSP7 sequences isolated from human hosts were obtained from GenBank at the National Center for Biotechnology Information. SARS-CoV (DQ022305, DQ071615, GU190215, KF367457, MG772933, MG772934, MN996532) and MERS-CoV (EF065505, EF065509) representative sequences isolated from bat hosts were also obtained from GenBank. NSP7 epitopes were compared across sequences obtained from isolates with fully sequenced alpha, beta, and epsilon genomes isolated from January 2020 to January 2021; gamma, delta, and lambda genomes isolated from January 2021 to August 2021; and omicron genomes isolated from November 2021 to March 2023. SARS-CoV-2 Wuhan-Hu-1 (GenBank id: MN908947) was selected as the reference strain.

### 2.2 T-cell epitope mapping

The SARS-CoV-2-NSP7 amino acid sequence was parsed into overlapping 9-mer sequences. Each 9-mer was assessed for its binding likelihood to a panel of nine HLA class II supertype alleles (HLA-DRB1*01:01, *03:01, *04:01, *07:01, *08:01, *09:01, *11:01, *13:01, and *15:01) using EpiMatrix version 1.3. Regions with high density of promiscuous putative T-cell epitopes were identified using ClustiMer. T-cell epitope clusters with scores ≥10 were selected for further analysis. This method is described in more detail in our previous publication (14).

### 2.3 T-cell epitope conservation analysis

T-cell epitope clusters were screened with JanusMatrix to identify epitopes cross-conserved with the human proteome as previously described (15). Epitopes were considered cross-conserved if they had identical TCR-facing residues (positions 2, 3, 5, 7, and 8) and were restricted by the same HLA-DRB1 alleles. Cross-conserved epitopes are more likely to engage cross-reactive T cells (16). For each T cell epitope cluster, a JanusMatrix homology score was calculated as the number of putative T cell epitopes in human proteins containing identical TCR-facing residues and restricted by the same HLA alleles as the epitopes predicted in the cluster, divided by the total number of epitopes in the cluster. This score indicates the average depth of coverage in the human proteome considering each T cell epitope in a cluster. Regions of high T cell epitope density with JanusMatrix homology scores above two were categorized as potentially either tolerated or actively regulatory.

Individual 9-mer frames of the SARS-CoV-2-NSP7 that were cross-conserved at TCR-facing residues with Tregitope 289 were also evaluated for conservation with highly pathogenic human and bat coronaviruses (SARS-CoV and MERS-CoV) and low-pathogenicity common cold coronaviruses (OC43, HKU1, NL62, and 229E).

### 2.4 Peptide synthesis

All peptides used in these studies (Tregitope FV621, 289, a shorter version of 289 named “289z” and NSP7-289) were synthesized by 21^st^ Century Biochemicals (Marlborough, MA). NSP7-289 was designed to include flanks on either side of putative binding frame for stability of the HLA-DR:peptide binding interaction. Two IgG-derived Tregitopes, 289 and 289z were synthesized for the purpose of comparing them to the NSP7 peptide in the HLA binding and immunomodulation assays described below. The Tregitope 289 peptide has previously been shown to induce bystander suppression to the tetanus toxoid (TT) antigen *in vitro* (12), to reduce T effector responses to co-administered antigens *in vivo*, and to expand Tregs *in vitro* and *in vivo* as measured by decreased proliferation of Teff cells and cytokine production (12, 13, 17, 18). Molecular weight accuracy was verified by mass spectrometry and all peptides were determined to be more than 90% pure by HPLC. Amino acid analysis was performed on all peptide samples to normalize net peptide content across assays.

### 2.5 HLA binding assays

NSP7-289 and control Tregitopes (289z, 289 and FV621) were tested for *in vitro* binding to HLA-DRB1*01:01, *03:01, *04:01, *07:01, *09:01, *11:01 and *15:01 alleles. HLA binding data for IgG Fc derived Tregitope peptides have been previously reported (19) and were repeated for comparison with NSP7-289. The HLA-DRB1 alleles selected for the HLA binding assays represent a wide array of class II alleles; each HLA-DRB1 allele functions as a “supertype allele” and shares similar binding peptide side-chain preferences for their binding pockets for the broader group of alleles (20).

EpiVax adapted the HLA binding assay originally described by Steere et al. (21) for *in vitro* validation studies. Peptides are solubilized with DMSO (Sigma Aldrich) in a stock solution for testing in a competition assay where the final DMSO concentration is negligible (0.0015 to 2%). Briefly, non-biotinylated test peptides, over a range of concentrations, are incubated with purified HLA-DR molecules (Benaroya Research Institute, Seattle, Washington) and a biotinylated, allele-specific competitor peptide of known HLA-DR binding affinity at a fixed concentration for that given labeled competitor peptide. A seven-point binding assay (0.1, 0.32, 1.0, 3.2, 10.2, 32.3 and 102.5 μM) was performed for each test peptide (NSP7-289, Tregitopes 289 and 289z). The binding assays were performed in triplicate for each of the concentrations. The percent inhibition values for each experimental peptide across a range of concentrations is used to calculate the IC_50_, the concentration at which 50% of the labeled control peptide’s specific binding is inhibited.

### 2.6 Human peripheral blood mononuclear cells

The Rhode Island Blood Center (RIBC) in Providence, RI provided leukocyte reduction filters from which PBMCs were isolated. Convalescent COVID-19 patients were recruited by Sanguine Biosciences (USA), a clinical service group that identified, consented, and enrolled participants. Subjects provided a written consent approved by the Institutional Review Board (IRB) SAN-BB-01. Additional de-identified convalescent COVID-19 donor PBMC were obtained from Dr. Daniel Hoft at Saint Louis University (Saint Louis University IRB Approval # 27790).

All samples were obtained in accordance with US Federal Policy for the Protection of Human Subjects (45 CFR 46).

Isolated PBMCs were then stored in liquid nitrogen until use for the experiments and the list of the donors are provided in **Supplementary Tables 1 and 2**. HLA class II haplotyping of donor PBMCs was performed using the One Lambda Micro SSPTM High Resolution HLA class II kit at the Hartford Hospital Transplant Immunology Laboratory (Hartford, CT) or using Sanger Sequencing-based HLA typing at the American Red Cross in Dedham, MA.

PBMCs isolated from healthy donors and convalescent COVID-19 donors were tested in the *in vitro* assays. The HLA alleles possessed by individual donors included in this panel represented HLA-DR alleles covering seven of the most prevalent HLA supertypes. These supertypes represent families of HLA-DR alleles that share similar binding preferences, covering >85% of the alleles found in human populations (22, 23). PBMCs were thawed and labeled with carboxyfluorescein succinimidyl ester (CFSE), and all assays were performed in RPMI complete media as previously described (12).

### 2.7 Tetanus toxoid bystander suppression assay

The Tetanus Toxoid Bystander Suppression Assay (TTBSA), which measures the inhibitory capacity of potential regulatory peptides on the recall response of human CD4+ T cells to the TT antigen, has been described in a previous publication (12). Since tetanus vaccination is a routine immunization, most donors PBMCs exhibit TT-specific memory T cells, which is required for these assays.

Briefly, PBMCs were labeled with CFSE (eBioscience) cell proliferation dye (2.5 μM) and then plated in RPMI complete media and rested overnight. The following day, test Treg epitope peptides (NSP7-289, Tregitopes 289 and 289z) were solubilized and added to the culture medium at a range of concentrations (8, 16, and 24 μg/mL) to demonstrate dose-dependent inhibitory capacity on TT-induced CD4+ T cell proliferation. Treg epitope peptide (FV621) was added as a positive control peptide at 24 μg/mL for the experiment. TT (Astarte Biologics, cat no. 1002) was then added to all wells at 0.5 μg/ml excluding media only (negative control) wells. Cells were cultured for 6 days, harvested at day 7, stained, and analyzed by flow cytometry.

### 2.8 CD8+ T cell recall response assay

To determine the effects of NSP7-289 on CD8+ T cell recall responses, and demonstrate that both CD4+ and CD8+ T cells can be suppressed by the Treg epitopes, PBMCs from healthy donors were stimulated with 0.5 µg/ml of a mixture of MHC class I peptides derived from cytomegalovirus, Epstein-Barr virus, and influenza virus designated CEF (CEF peptide pool, Mabtech, cat no. 3615-1), alone or with the of test peptides. In addition to NSP7-289, additional known regulatory peptides (Tregitopes 289, 289z and FV621) were included for comparison. PBMCs were cultured as described above in the TTBSA. As described in previous studies (12), CEF-specific CD8^+^ T cell proliferation was measured in the presence or absence of test peptides after six days of incubation by flow cytometry analysis of CFSE dilution.

### 2.9 Antigen-specific CD4+ T cell recall response assay

To assess the effects of NSP7-289 on CD4+ T cell recall responses in COVID-19 convalescent donors, PBMCs from convalescent donors were stimulated and cultured as described above in TTBSA with 5.0 µg/ml of CPI Positive Control Solution, a mixture of protein antigens from the cytomegalovirus, influenza, and parainfluenza (ImmunoSpot, Cat# CTL-CPI-001), alone or with the addition of test peptides. As described previously (12), antigen-specific CD4+ T cell responses were measured in the presence or absence of test peptides after six days of incubation, cells were stained with surface markers and CD4+ T cell proliferation analyzed by flow cytometry.

### 2.10 Flow cytometry and gating strategy

As previously described, cells were washed, stained with Live/Dead aqua fixable stain and with surface receptor antibodies (12). The CD3 (OKT3)-PECy7, CD4 (OKT4)-BV410, and CD8 (SK1)-APC antibodies used in this assay were purchased from BioLegend and the CFSE proliferation dye was purchased from eBiosciences. Data acquisition was performed using the Attune NxT Cytometer (Life Technologies) and analyzed with FlowJo software version 10.9 (Treestar, Inc). Viable lymphocyte populations were first identified by a low forward scatter (FSC) and low side scatter (SSC) gate followed by doublet exclusion by plotting the height and width versus the area of side and forward scatter and dead cells were gating out by Live-Dead staining.

For analyzing proliferation of CD4^+^ and CD8^+^ T cell populations, lymphocytes were first gated on either CD3^+^CD4^+^ or CD3^+^CD8^+^ cells and their proliferation was evaluated by a decrease in the CFSE signal typically seen in a proliferating population identified as CFSE^low^.

### 2.11 Statistical analysis

Prism software (GraphPad version 8.3) was utilized for the statistical analysis. Unless otherwise indicated, the Welch’s t-test (unpaired, two-tailed) was utilized to attain the significance of differences between the antigen-only (e.g., TT, CEF, CPI) stimulated cells to the antigen + test peptide treated cells (e.g., TT + Tregitope 289z). Differences were considered significant when *p* < 0.05 (*), very significant when *p* < 0.01 (**), highly significant when *p* < 0.0002 (***), and extremely significant when *p* < 0.0001 (****).

## 3 Results

When SARS-COV-2 emerged, our group used immunoinformatic tools included in the iVAX toolkit (EpiMatrix, ClustiMer and JanusMatrix, EpiVax, Providence RI (14)) to search for epitopes for COVID-19 vaccine design. The identified epitopes were screened for potential tolerogenic epitopes as a matter of routine (See iVAX publication (14) for rationale). Surprisingly, we identified a Tregitope-like sequence in the virus’ NSP7 protein, which is a close homolog of the IgG Fc-derived Tregitope 289 (NSP7-289).

**Figure 1:**
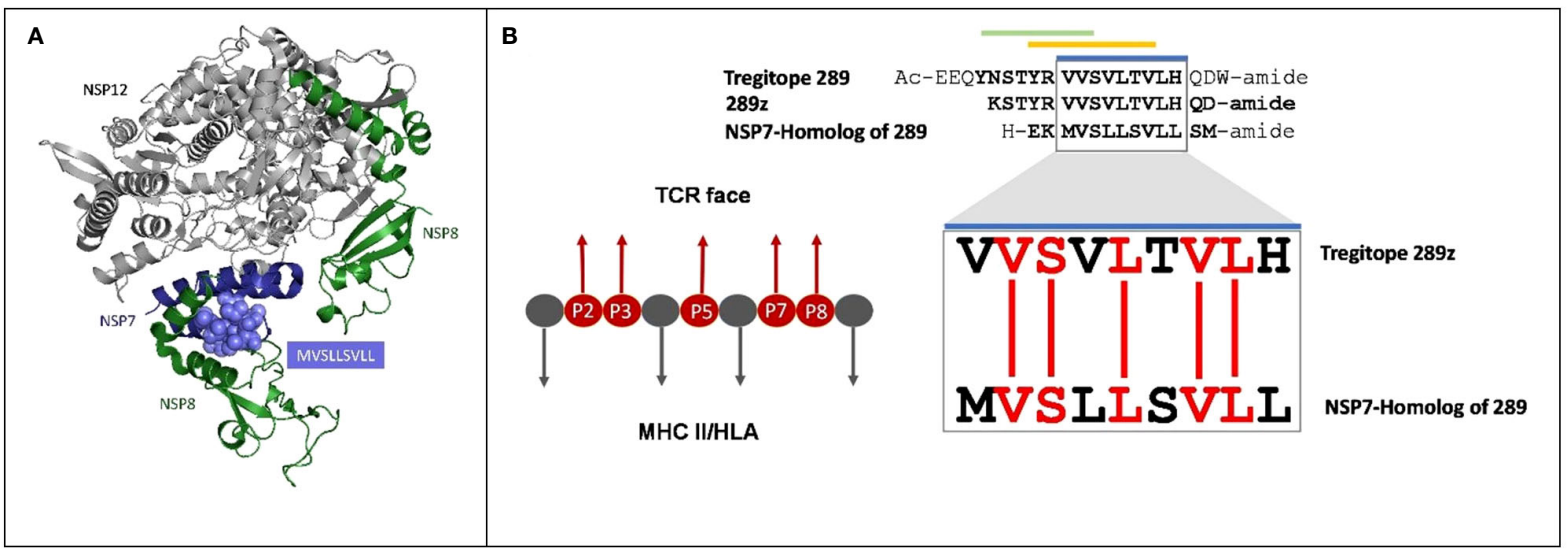
Cross-conservation of the NSP7 predicted T cell epitope with Tregitope 289. **(A)** NSP7 shown in the context of the RNA-dependent RNA polymerase structure where it forms a complex with two NSP8s and one NSP12 (pdb id 7BV1) (24). NSP7 peptide residues are represented as purple spheres (MVSLLSVLL) within the NSP7 ribbon structure. Graphic was adapted from structure of the nsp12-nsp7-nsp8 complex obtained from the PDB (https://www.rcsb.org/ on Sept 5th, 2023). **(B)** Alignment of the human Tregitope peptide sequences Tregitope 289 and 289z to the NSP7-homolog sequence. The TCR-facing residues in the HLA binding pocket (P2, P3, P5, P7 and P8) of Tregitope 289z are shown in red and are 100% conserved with NSP7.

NSP7-289 is conserved with one of the promiscuous nine-mer sequences known as an EpiBar in Tregitope 289 (289z sequence). The Tregitope 289z sequence is located in the C-terminal region of the extensively published Tregitope 289 from the IgG Fc domain (see **Figure 1B**). NSP7-289 and Tregitope 289z have predicted HLA binding epitopes and identical T cell receptor (TCR)-facing residues. These amino acids face towards the T cell receptor, outside the HLA binding cleft, and are accessible to the host’s TCR repertoire. T cell receptors usually only interact with the surface-facing amino acid side chains and may not ‘see’ differences in the HLA-binding amino acid sequence, which is why similar binding epitopes that have identical TCR-facing residues may stimulate the same T cell clone (15, 25).

NSP7-289 was synthesized and evaluated in several *in vitro* assays including an HLA binding assay and several T cell bystander suppression assays that utilized different antigens (TT, CEF and CPI). These assays confirmed the HLA-DR binding predictions and demonstrated that NSP7-289 performed similar to other well defined regulatory T cell epitopes.

### 3.1 Identification of NSP7-289: A SARS-CoV-2 T cell epitope cluster homolog to Tregitope 289

SARS-CoV-2 proteins from the reference Wuhan-Hu-1 strain were screened for potential CD4+ T cell epitopes using EpiMatrix (26). For each protein, T cell epitope clusters defined as regions with high HLA class II T cell epitope density predicted to bind multiple HLA-DRB1 supertype alleles were identified. Each cluster was assessed for its potential to induce regulatory T cell responses using JanusMatrix, which screens for epitopes in the human proteome with identical TCR-facing residues that are predicted to bind the same HLA-DRB1 alleles as those within a given epitope cluster (i.e., cross-conserved epitopes). JanusMatrix human homology was assessed by calculating the average depth of homology in the human proteome. Clusters with significant human homology were those with scores above two.

Based on the T cell epitope prediction and JanusMatrix analysis, one cluster from SARS-CoV-2 NSP7 (50-62) (**Table 1**) was identified. One 9-mer frame from the NSP7 cluster (F52: MVSLLSVLL) was cross-conserved with the TCR-face of the C-terminal epitope within Tregitope 289 (Tregitope 289z; **Figure 1B**). In addition, the NSP7 Tregitope homolog sequence was found to be conserved in >99.7% of 1,046,953 SARS-CoV-2-NSP7 sequences. This 9-mer frame was also highly conserved (100% identity) among other beta coronaviruses, including human and representative bat NSP7 sequences (**Table 2**). NSP7 frame F53 (VSLLSVLLS) was also highly cross-conserved with human epitopes (**Table 1**) and highly conserved among SARS-CoV-2 and human and bat SARS-CoV sequences (**Table 2**).

**Table 1:** T cell epitope and cross-conservation analysis of the NSP7 Tregitope 289 homolog.

Antigen	Cluster address	Cluster sequence	EpiMatrix HLA-DRB1 hits (predicted epitopes)	HLA-DRB1 alleles with EpiMatrix hits	EpiBars	EpiMatrix cluster score	JanusMatrix human homology score
NSP7	50-62 **(F50)**	EKMVSLLSVLLSM	16	9	2	31.97	11.0
	52-60 **(F52)**	MVSLLSVLL	9	9	1	21.11	6.89
	53-61 **(F53)**	VSLLSVLLS	6	6	1	11.83	16.17

**Table 2:** Conservation (100% identity) of NSP7 Tregitope 289 homolog in coronaviruses.

Coronavirus	SARS-CoV-2 alpha	SARS-CoV-2 beta	SARS-CoV-2 gamma	SARS-CoV-2 delta	SARS-CoV-2 epsilon	SARS-CoV-2 lambda	SARS-CoV-2 omicron	hu SARS-CoV	hu MERS-CoV	hu CoV-HKU1	hu CoV-OC43	hu CoV-NL63	hu CoV-229E	bat SARS-CoV	bat MERS-CoV
Number of sequences	18,337	2,322	59,684	339,829	1,980	4,185	620,616	155	364	1	171	38	6	7	2
Conservation frame 52-60	99.89%	99.35%	99.78%	99.67%	100%	99.43%	99.85%	100%	0%	0%	0%	0%	0%	100%	0%
Conservation frame 53-61	99.89%	99.35%	99.78%	99.68%	100%	99.43%	99.86%	100%	0%	0%	0%	0%	0%	100%	0%

**Figure 2:**
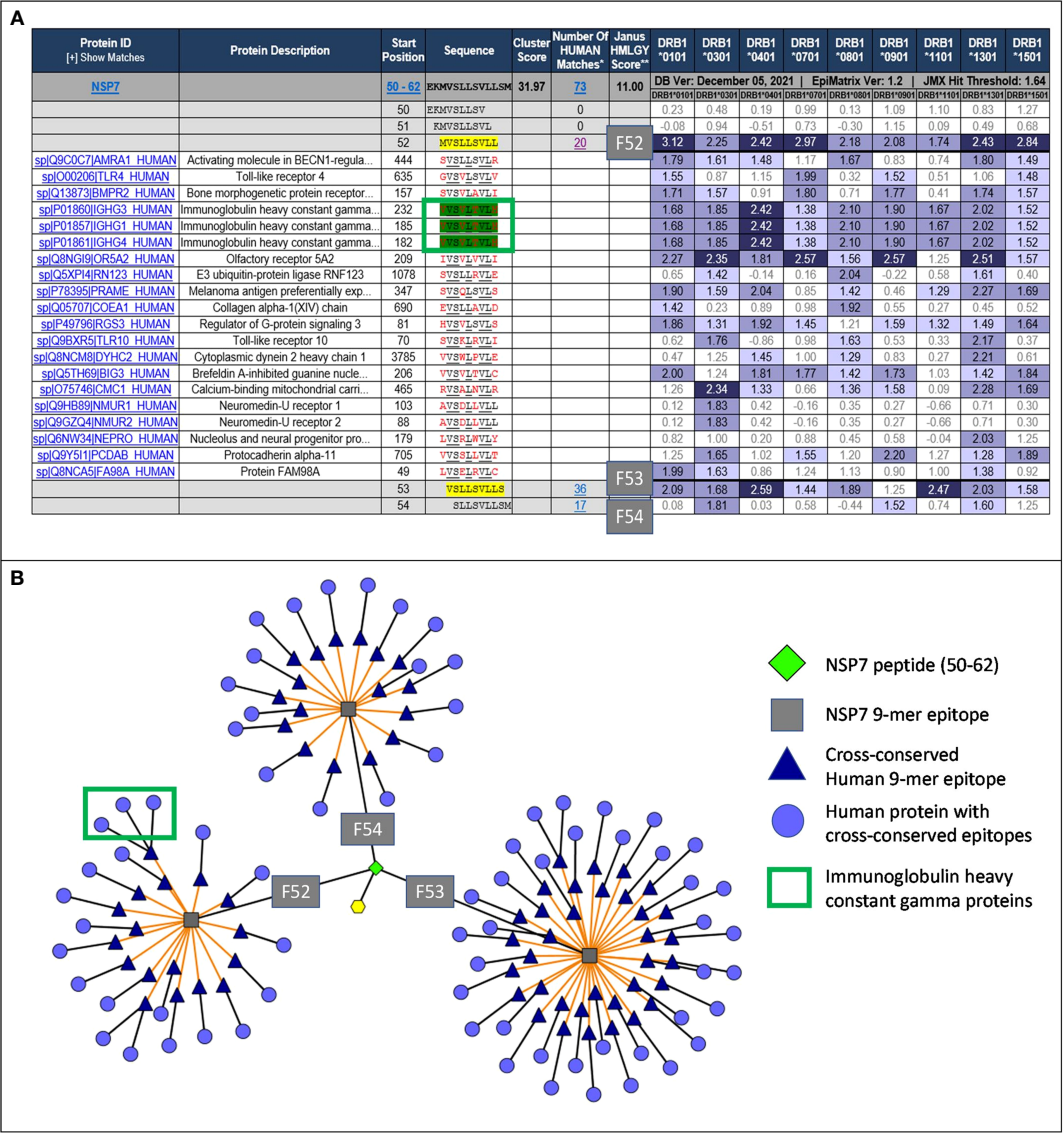
JanusMatrix analysis and TCR-Epitope Network for NSP7. **(A)** JanusMatrix analysis showing the cross conservation of SARS-CoV-2-NSP7 peptide with human proteins for Frame 52 (F52). The green box highlights the epitope in NSP7 containing TCR residues (underlined) cross-conserved with the Tregitope 289 sequence within the IgG1 constant region. See **Supplement Figure S1** for a fully expanded JanusMatrix analysis for all three NSP7 9-mer epitopes (F52, F53 and F54). **(B)** Cytoscape of JanusMatrix analysis showing the TCR-Epitope network of SARS-CoV-2-NSP7 peptide and its TCR cross-conservation with other human proteins. F52, F53, and F54 denote the 9-mer frames in the NSP7 sequence that contain epitopes that are highly cross-conserved with human epitopes, as illustrated in this figure.

**Figure 1A** shows the protein ribbon model of the RdRp complex of SARs-CoV-2 containing NSP12, NSP7 and two copies of NSP8. NSP7 is important to the formation and functions of RdRp complex, and studies have shown that mutations in NSP7 may play a role in the stabilization of the RdRp complex (27). The JanusMatrix cross-conservation results for the NSP7 epitope to the human proteome using Cytoscape (28) to create visualized TCR-Epitope Network, illustrates how the source protein, NSP7 and the NSP7 epitopes are mapped to TCR-matched epitopes in the human proteome database and linked to multiple human proteins (**Figure 2B**).

JanusMatrix analysis reveals that three putative nine-mer frames (F52, F53, and F54) within the NSP7 peptide (50–62) sequence are cross-conserved at the TCR-face with 73 human proteins and their corresponding human T cell epitopes (**Figure 2A**). The overall JanusMatrix homology score for the NSP7 peptide is 11.0, whereas homology scores above 2 in pathogenic sequences are likely to have a higher degree of human-like epitopes (or humanness) and may be tolerated and/or potentially induce regulatory T cells (Tregs) in their host.

The 9-mer epitope in frame 52 [F52] was the primary focus of this study, since this epitope is cross-conserved to three highly prevalent human Immunoglobulin heavy chain proteins (IgG1, IgG3 and IgG4), is predicted to bind to nine HLA-DRB1 supertypes impacting >90% of the global population, has the highest binding potential as indicated by the EpiMatrix score (**Table 1**) and is cross-conserved with a well-characterized Tregitope 289 shown to have regulatory properties in both *in vivo* and *in vitro* models (12, 18). Based on these characteristics we hypothesized that the F52 epitope in NSP7 may exhibit regulatory T cell activation properties similar to Tregitope 289, and thereby engages immunosuppressive Tregs and potentially promote host tolerization to other associated SARS-CoV-2 proteins (e.g., RdRp complex).

### 3.2 NSP7-289 binds multiple HLA-DRB1 alleles

**Figure 3:**
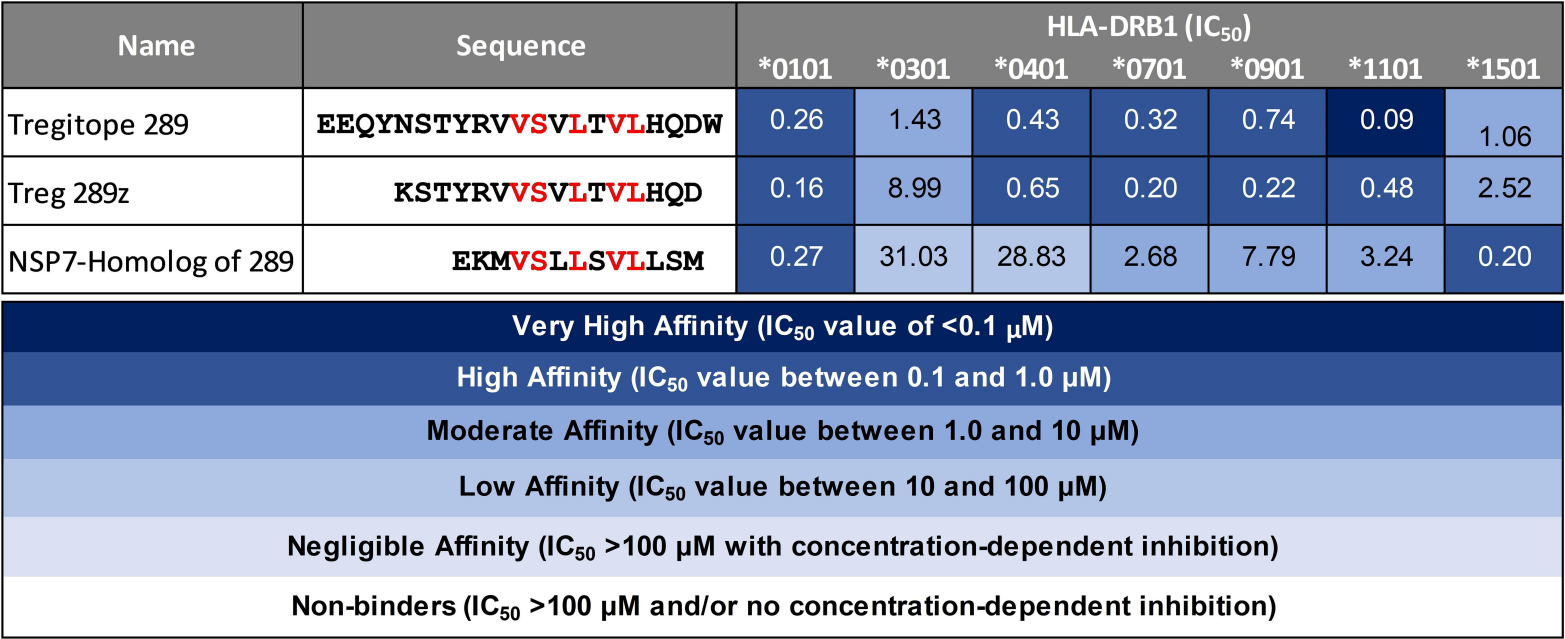
HLA-DRB1 binding of SARS-CoV-2-NSP7 peptide alongside IgG Tregitopes 289 and 289z for comparison. Selected SARS-CoV-2-NSP7 peptide was evaluated for HLA-DRB1 binding *in vitro* and the calculated IC_50_ values are shown. The SARS-CoV-2-NSP7 peptide exhibited binding across multiple alleles tested (DRB1*01:01, *03:01, *04:01, *07:01, *09:01, *11:01, and *15:01). A seven-point competition assay using a validated control peptide was performed; color coding reflects binding affinity. IC_50_ was determined by interpolation. IgG Tregitope 289 and 289z also bind to all of the tested alleles.

Peptide presentation by major histocompatibility complex (MHC, also known as HLA in humans) proteins on the surface of antigen presenting cells (APC) plays a pivotal role in the T cell response to pathogens. To evaluate the possibility of SARS-CoV-2 NSP7 peptides being presented to regulatory T cells, measurements of peptide binding to various HLA-DRB1 molecules were performed. The SARS-CoV-2-NSP7-homolog of 289 peptide showed low to high affinity binding to the panel of HLA alleles evaluated in this assay, as reflected by the half maximal inhibitory concentration (IC_50_) values calculated from the dose-response curve (**Figure 3**). Similarly, both the 289 and 289z peptides demonstrated binding to all of the tested HLA-DRB1 alleles (HLA-DRB1*01:01, *03:01, *04:01, *07:01, *09:01, *13:01, and *15:01) with a moderate to high affinity. Despite differences in HLA-facing positions compared to Tregitope 289(z), the binding of the SARS-CoV-2 NSP7 homolog of the 289 peptide across multiple HLA-DRB1 alleles is comparable, and since their TCR-facing residues are identical, the NSP7 homolog therefore may be recognized by Tregitope 289-specific T cells. Moreover, the NSP7 Tregitope homolog has the potential to induce Treg response in a significant portion of the human population due to its binding affinity to a wide range of HLA-DRB1 alleles.

### 3.3 NSP7-289 suppressed CD4+ T cell proliferation in a Tetanus Toxoid recall response

The Tetanus Toxoid Bystander assay (TTBSA) was used to determine the effect of NSP7-289 on effector T cell suppression and TT-specific CD4+ T cell proliferation (12). PBMCs from healthy donors generate memory CD4+ T cell recall response to TT by upregulating several activation markers on the cell surface as observed by flow cytometry. Assuming that most of the donors were vaccinated with TT, and depending on the TT-specific precursor memory CD4+ T cells in the donor blood, CD4+ T cell recall responses vary by 5-60% in different donors (12), likely related to the TT vaccination status of the donors.

**Figure 4:**
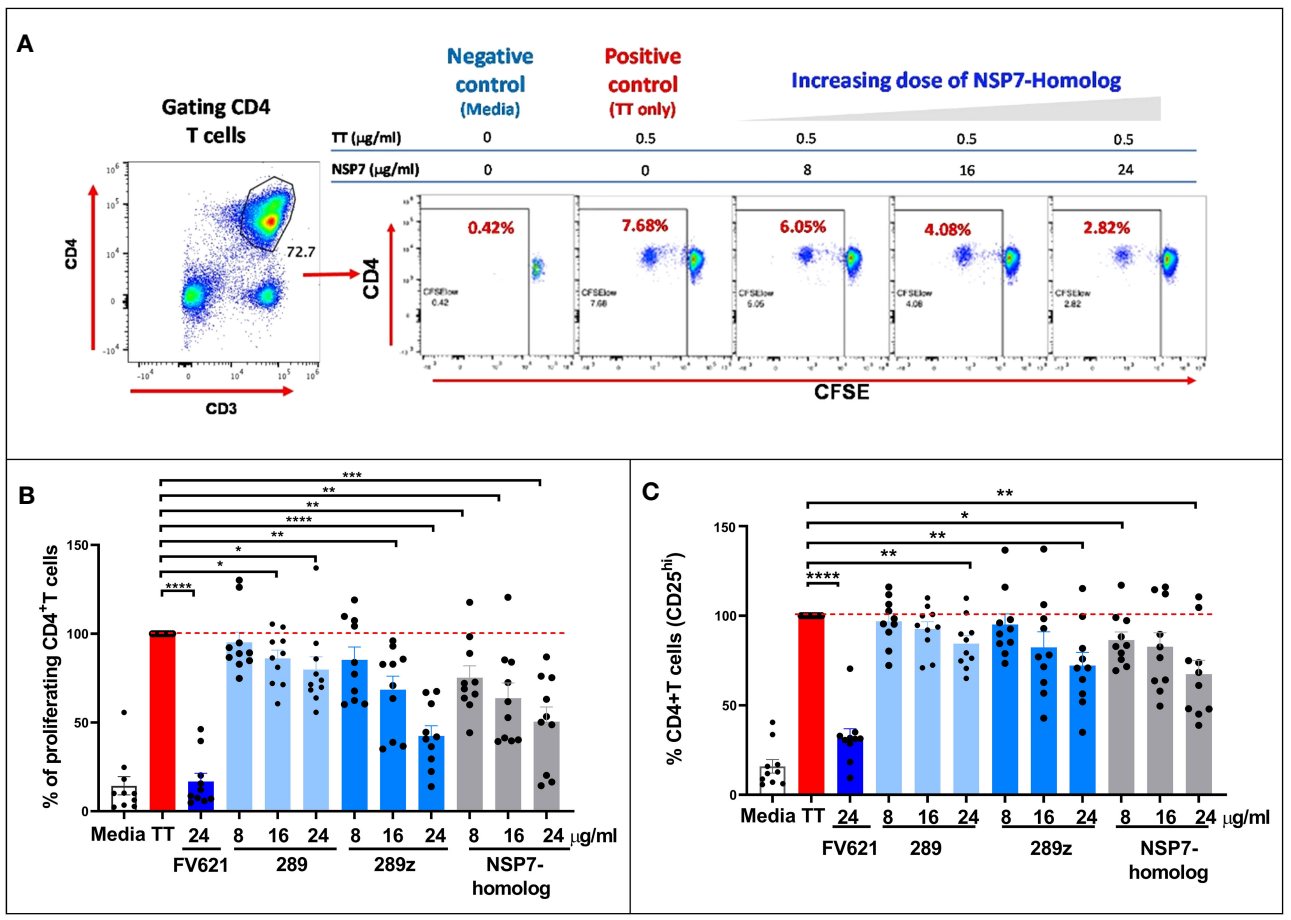
Comparison of the inhibition of CD4+ recall response by SARS-CoV-2-NSP7-homolog of 289 peptide with IgG-derived Tregitopes 289 and 289z in the TTBSA. PBMCs from healthy donors were stimulated with 0.5 µg/ml of TT alone or with FV621 or increasing concentrations of 289, 289z or SARS-CoV-2-NSP7 peptide (8, 16 and 24 μg/ml). Proliferation of CD4+ T cells was measured six days post-stimulation by flow cytometry using a CFSE dilution parameter. **(A)** Left panel shows the gating of CD4+ T cells. See **Supplement Figure S2** for the detailed gating strategy. The right panel shows representative flow plots for one donor indicating the dose effect on the proliferation of CD4+ T cells with increasing concentrations of SARS-CoV-2-NSP7 peptide. **(B, C)** Graphs show the percent of CD4+ T cell proliferation **(B)** and the percent of CD4+ T cell activation (CD25^hi^ cells) **(C)** for each peptide tested in the assay, compared to TT stimulation alone. FV621 was used as a positive control for suppressed proliferation (12). Data in the graphs are normalized to TT stimulation at 100% proliferation or activation (0% suppression), and the percent suppression by each concentration of each test peptide can be determined by comparison. Data shown are the cumulative results of 10 donors. P values * = <0.05, ** = <0.01, *** = <0.0002 and **** = <0.0001 represent statistical significance between Tregitope stimulation *vs*. TT using a two-tailed t test.

The capacity of NSP7-289 to inhibit the effector response to TT in PBMCs derived from a panel of 10 healthy donors (**Figure 4**) was evaluated. The previously characterized FV621 peptide (12) was also included as a positive control Tregitope. This peptide has shown high binding affinity for multiple HLA-DRB1 alleles and robust suppressive capacity in the bystander T cell assays.

As shown for one representative donor in **Figure 4A**, TT stimulation increased CD4+ T cell proliferation by roughly 20-fold in a CFSE dilution assay (see flow cytometry dot plot for the positive control “TT only”). The addition of NSP7 Tregitope suppressed proliferation of CD4+ T cells to TT in a dose-dependent manner. These data indicate that treatment of TT stimulated PBMCs with NSP7-289 inhibits CD4+ T cell proliferation.

Both Tregitope 289 and the shorter version of Tregitope 289 known as 289z were tested in this assay, based on their shared TCR facing residues and observed binding affinity to HLA-DRB1 alleles. The inhibitory capacities of these peptides were comparable in the TT-bystander suppression assay at similar doses. **Figure 4B** shows that Tregitope peptide 289z inhibited TT-mediated CD4+ T cell proliferative response in a dose-dependent manner. Like 289z, NSP7-289 also inhibited CD4+ memory T cell proliferation upon TT-stimulation. FV621 is used as a positive control Tregitope and has a greater suppressive effect on CD4+ T effector memory cells proliferation (12) *in vitro* shown in **Figure 4B**. These data indicate that the newly identified Tregitope NSP7-289 also suppressed the activation of CD4+ T cells, as measured by the reduction of CD4 T cell frequency expressing activation marker CD25, in a dose dependent manner, similar to IgG Tregitope 289z (**Figure 4C**).

**Figure 5:**
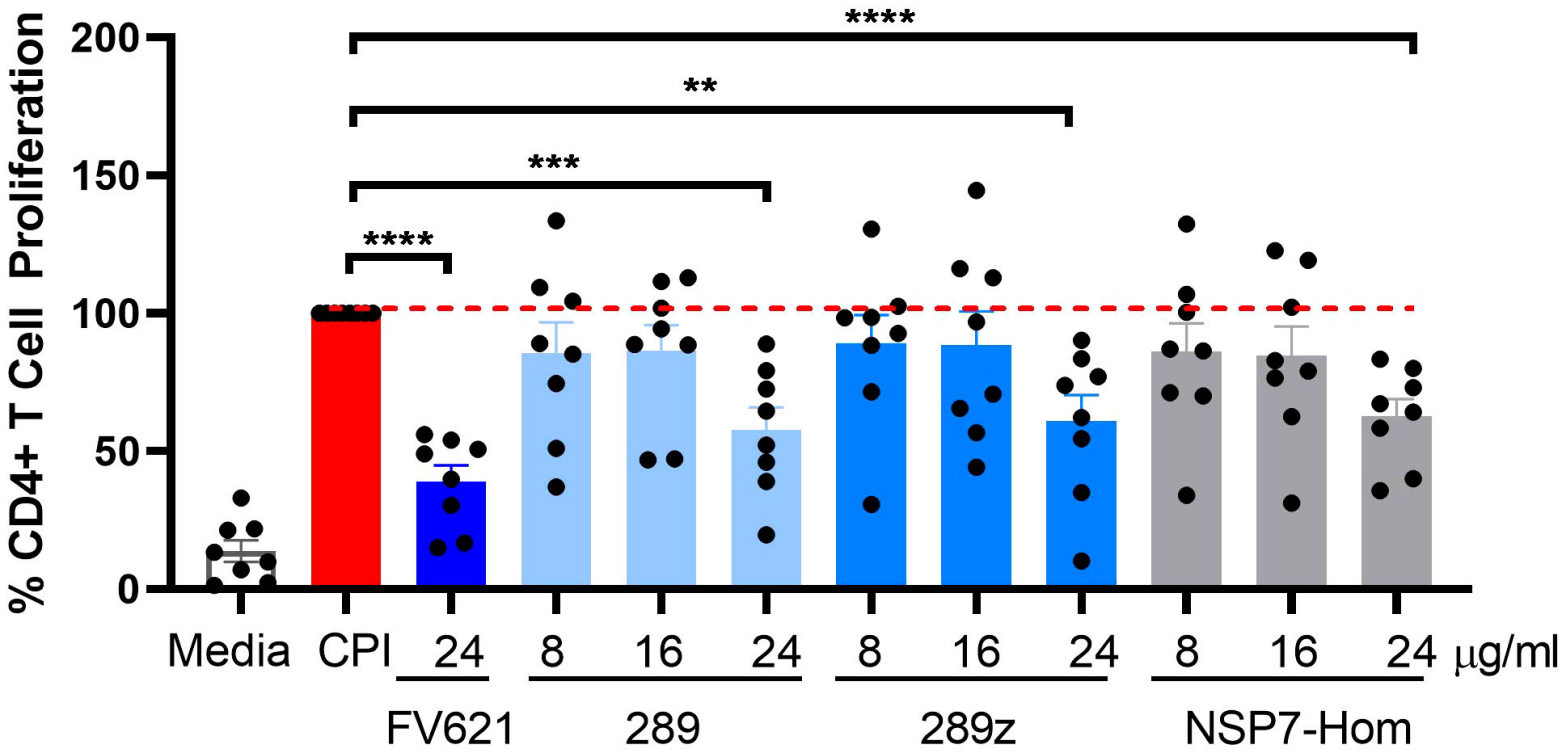
Inhibition of antigen-specific CD4+ recall response by SARS-CoV-2-NSP7-homolog of 289 peptide. PBMCs from convalescent donors were stimulated with 5.0 µg/ml of CPI alone or with increasing concentrations of 289, 289z or SARS-CoV-2-NSP7 peptide (8, 16 and 24μg/ml) for 7 days. FV621 was used at 24μg/ml for positive control of the experiment. Proliferation of CD4+ T cells was measured at day 7 by flow cytometry using a CFSE dilution parameter. Data in the graph are normalized to CPI stimulation at 100% proliferation and combined from two experiments. Data shown are the cumulative results of 8 donors. P values ** = <0.01, *** = <0.0002 and **** = <0.0001 represents statistical significance between Tregitope stimulation *vs*. CPI using a two-tailed t test.

The inhibitory effect of the NSP7-289 on CD4+ T cell proliferation was also tested using PBMCs collected from COVID-19 convalescent donors. The CD4+ T cell activating CPI (a mixture of protein antigens from cytomegalo-, influenza and parainfluenza viruses) was used as an alternative antigen to TT. It has been shown that CPI induces the production of IFNγ specifically by CD4+ T cells (29). CPI-induced CD4+ T cell proliferation in convalescent donor PBMCs was observed at day seven in culture. Co-incubation of CPI with Tregitopes (FV621 or 289z) suppressed CPI-induced CD4+ T cell proliferation (**Figure 5**). NSP7-289 similarly suppressed CD4+ T cell proliferation, indicating the identification and validation of a Tregitope sequence in SARS-CoV-2.

### 3.6 Inhibition of CD8 T cell response by NSP7-289

Interactions between MHC-class I specific peptides and CD8+ T effector memory cells can result in the expansion of CD8+ T effector memory cell populations. However, Tregitope activated Tregs can mediate an inhibitory effect on activated CD8+ T cells (26) through the upregulation of cell surface inhibitory receptors on dendritic cells. The inhibitory effect of NSP7-289 on the CD8+ T cell responses to a pool of cytomegalovirus, Epstein-Barr virus and influenza virus-derived MHC class I peptides was also evaluated, using a standardized reagent called CEF, in PBMCs from healthy donors following a protocol essentially identical to the one used to evaluate the CD4+ T cell response in TTBSA (see Methods).

**Figure 6:**
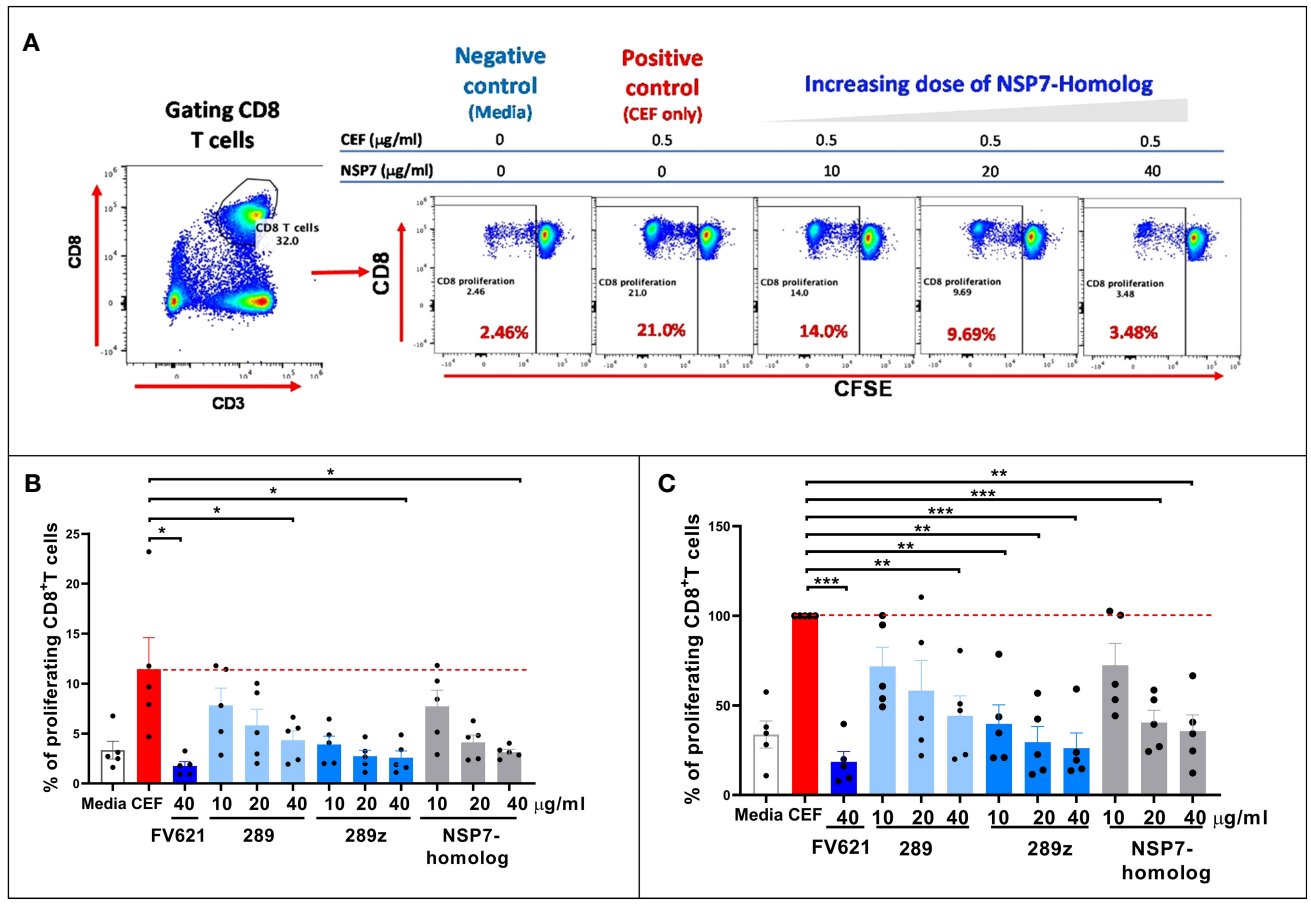
Effect of SARS-CoV-2-NSP7 peptide on CD8+ T cell response in PBMCs from healthy donors stimulated with CEF peptides. PBMCs from healthy donors were stimulated with 0.5 µg/ml of CEF alone or in combination with FV621 or increasing concentrations of 289, 289z or SARS-CoV-2 NSP7 peptide (10, 20 and 40μg/ml). Proliferation of CD8+ T cells were measured six days post-stimulation by flow cytometry based on CFSE dilution. **(A)** Left panel shows the gating of CD8+ T cells. In the right panel, representative flow plots display the dose effect on the proliferation of CD8+ T cells resulting from increasing concentrations of SARS-CoV-2-NSP7-homolog peptide. **(B, C)** Graphs show the (%) proliferation of CD8+ T cells for each peptide tested in the assay, compared against CEF stimulation alone so that percent inhibition can be calculated or inferred. FV621 was used as a positive control as it has demonstrated consistent suppression of CEF response across donors in our previous publication (12). Original data is presented in panel B, and data normalized for CEF stimulation as 100% proliferation in panel C. The percent inhibition of CD8+ T cell proliferation for each peptide at each concentration tested in the assay compared to CEF stimulation alone. Data are combined from five donors in the experiment. P values * = <0.05, ** = <0.01 and *** = <0.0002 represent statistical significance *vs*. CEF stimulation alone using a two-tailed t test.

As shown in **Figure 6A**, flow cytometry data from a representative donor shows that CD8 T cells from healthy donor PBMCs proliferated upon stimulation with CEF as determined by CFSE dilution. Co-stimulation of NSP7-289 with CEF strongly inhibited CD8+ T cell proliferation (CFSE^low^) response to CEF stimulation in a dose-dependent manner. **Figure 6B** shows the unnormalized data measuring percent proliferation of CD8+ T cells by individual Tregitopes whereas **Figure 6C** shows the same data normalized to CEF stimulation as 100% proliferation (0% inhibition). Normalization to eliminate the impact of donor-specific CEF responses shows a significant inhibitory effect of NSP7-289 on CD8+ T cells proliferation. The notion that competition for HLA binding on the APC between the Tregitope and the CEF peptide pool may lead to this inhibitory effect is precluded since the binding of Tregitope to MHC class II cannot interfere with CEF peptide binding to MHC Class I. These data also suggest that NSP7-289 can modulate both CD4+ and CD8+ effector T cell responses.

## 4 Discussion

In this project, *in silico* tools were used to identify a putative regulatory T cell epitope in SARS-CoV-2 and the inhibitory effect of NSP7-289 was explored *in vitro*. In previous studies, Tregitope-like sequences in pathogens were shown to engage natural human Tregs and reduce antibody responses *in vivo*. Our work has also demonstrated that removal or modification of these Tregitope-like sequences improves the antigenicity and efficacy of certain vaccines. For example, removal of a Tregitope-like sequence identified in H7N9 influenza hemagglutinin improved protection against influenza virus when the modified vaccine was used to immunize humanized mice (HLA-DR3) that were the challenged with live H7N9 (30).

There may be ample opportunity for pathogens to camouflage themselves against immune response to critical antigens, as Tregitope-like peptides are not uncommon in the human genome, Several cross-conserved CD4+ T helper (HLA class II binding) Tregitope-like epitopes that are present in other human proteins have also been shown to be actively tolerogenic (via activation of regulatory T cells) (13, 17). For example, we recently identified and validated a Tregitope, FV621, which is found in the sequence of factor V, a highly prevalent serum protein (12). Other putative Tregitope peptides include the “Edratide” peptide of human CDR1 (complementarity-determining region 1) (31), which is similar to Tregitope 029, and heat shock protein 70 (HSP70)-derived peptide B29 (32). These tolerance-inducing epitopes share key features with established Tregitopes, such as promiscuous HLA-DR binding, presence in the sequences of highly prevalent human proteins and cross-conservation across mammalian species.

The exact role of these naturally occurring Tregitopes in the human proteome is unknown. They may play an important role in the control of auto-reactivity (33, 34).Tregitopes, including Tregitope 289, have been shown to promote antigen-specific tolerance in murine models of autoimmune diseases including type 1 diabetes (17, 35–37), inflammatory bowel disease (36), and multiple sclerosis (experimental autoimmune encephalomyelitis) (37), as well as models of gene therapy (38) and allergy (18, 39). IgG Tregitope-specific natural Tregs (nTregs) modulate T effector responses by inhibiting the activity of autoreactive effectors and/or by converting the phenotype of T effector to induced Tregs (iTregs) as shown in tolerization to OVA in DO11.10 mice (17) and in HLA TCR-transgenic mice in a skin transplant model [ref (40)]. Several of the non-IgG Tregitopes have been found to be effective in murine models of lupus (41), rheumatoid arthritis and collagen-induced arthritis (42).

Similarly, the exact role of the Tregitope-like sequence in NSP7 is unknown. NSP7 of SARS-CoV-2 forms a complex with two NSP8 molecules and one NSP12 molecule in the RNA-dependent RNA polymerase (RdRp) complex. The α-helical structure of NSP7, together with NSP8, plays a role in the stabilization of the NSP12 regions involved in RNA binding, which is essential for a highly active NSP12 polymerase (43–45). The RdRp complex recognizes and processes RNA from the virus for ribosomal transcription of viral proteins and virus propagation. In the absence of NSP7, the NSP12 polymerase engages only one NSP8 and does not bind RNA, making NSP7 essential for stability of the RdRp complex (45). RdRp, which is responsible for viral RNA replication in host cells, is a promising target for therapy as there is no host cell homolog. The nucleotide analog remdesivir, which is the only drug for COVID-19 that targets the RdRp, was approved for emergency use in 2021 (46).

NSP7 is found in the RNA-dependent RNA polymerase of SARS-CoV-2. NSP7-289 strongly inhibited both CD4+ and CD8+ T cell memory responses with low to high affinity to multiple class II HLA-DR alleles. Similar to Tregitope 289z, NSP7-289 might influence human immune responses to SARS-CoV-2 *in vivo* in a significant proportion of the population. NSP7-289 appears to have immune modulating properties that are similar to known Tregitopes 289 and 289z, as it is capable of down-modulating both memory CD4+ and CD8 T effector responses in bystander suppression assays. The effect was observed in both healthy and convalescent donors, indicating the importance of NSP7-289 identification while showcasing the potential importance of regulatory epitopes present in SARS-CoV-2.

As it is difficult to expand regulatory T cells and to identify their TCR identities, it is possible that we have misidentified the most tolerogenic region of this T cell epitope. The epitope found in Frame 53 that is predicted to bind to six HLA-DRB1 supertypes and has 36 human protein matches may be contributing to the tolerogenicity of the peptide sequence found in NSP7 (**Table 1**). Additional de-convolution of the tolerogenic response to the NSP7 sequence containing the epitopes may be important to perform before the sequence is modified or removed (to improve the efficiency of a vaccine containing NSP7) at some point in the future.

One potential explanation for the persistence of the Treg epitope in NSP7 may be that it is important for the protection of NSP7 against immune response by T cells. RNA-dependent RNA polymerase (RdRp) is a mutation hotspot in SARS-CoV-2. The non-structural protein 7 (NSP7) forms a complex with two NSP8s and one NSP12 to form the RdRp replication machinery for viral genome replication and viral protein translation. Therefore, putative T regulatory epitopes occurring in the NSP7 protein may play an important role in the protection against immune responses to the replication machinery complex, by tolerizing its host against the RdRp and new mutants arising in this complex. One of the first mutational hot spots in SARS-CoV-2 was found in NSP12, part of the RdRp complex, and reported in February 2020 after analysis of the virus isolated from Europe (47). Quite different from NSP12 sequence, the epitope within NSP7-289 was found to vary in only 0.2% of strains of SARS-CoV-2 while there is little overall variance in this sequence in > 1 million analyzed sequences. The appearance of the Tregitope homolog in SAR-CoVs and its persistence throughout the evolution of SARS-CoV-2 since the virus first emerged seems to indicate that the presence of a Treg epitope in the sequence of the protein may be critically important for viral survival.

Pathogens escape host defenses by T-cell epitope mutation or deletion and by simulating the appearance of human T cell epitopes. These pathogen ‘behaviors’ are known as immune escape and immune camouflage, respectively (7, 8, 48). The purpose of this study was to determine whether the Tregitope-like sequence in NSP7 possessed similar tolerogenic properties to Tregitope from human IgG. While the presence of this epitope in SARS-CoV-2 may be serendipitous, it may still have an impact on immunogenicity, and the sequence may play a role in the preservation of the function of the NSP7 gene in SARS-CoV-2 and beta coronaviruses.

And while Treg suppression of Class II T helper cells (that promote antibody responses) may not be relevant to the protection of internal proteins, activation of Tregs may also modulate class I restricted T cell responses. The independent effect of Tregitopes on Class I-restricted T cell responses was also observed using individual virus-derived, MHC Class I-restricted epitopes in a previous publication (16). This effect is possibly due to secretion of suppressor cytokines and/or modulation of cell surface inhibitory receptors, leading to generalized suppression regardless of target T effector cell type (CD4+ or CD8+), as suggested by the previous observation that Tregitope-stimulated PBMCs were suppressive in trans-well plates (27).

This report supports previous findings that Treg epitopes may be present in the protein sequences of pathogens. While it is impossible to prove that the SARS-CoV-2 pathogen may have ‘evolved’ to present this epitope, other pathogens may develop what has been called ‘immune camouflage’ to reduce the potential immune response in their human host. Pathogens escape immune surveillance by interfering with both innate and adaptive immune responses through a variety of mechanisms. Treg responses provide another means of immune escape. Treg responses have also been identified during influenza (49), malaria (50), and *Trypanosoma cruzi* infection in humans, in leishmaniasis (51), and in a demyelinating disease of mice caused by coronavirus (52). Regulatory T cells may contribute to “disease tolerance” which may be critical to human survival of parasite infection and viral diseases, as described by Martins et al. (53).

Several studies have identified a role for regulatory T cells in the exacerbation of COVID-19 disease in humans (54, 55). The epitope targets of the regulatory T cell response were not identified in these studies. Identification of the regulatory T cell epitopes in self-antigens and detection of cross-reactive pathogen epitopes may clarify the connection between ‘immune camouflage’ and ‘epitope mimicry’ that is associated with autoimmune disease (55).

Liise-Anne Pirofski and Arturo Casadevall have proposed the involvement of the “damage-response framework” in infectious disease, wherein the intensity of the host response may modify the manifestation of an infectious disease (56). For some individuals, severe inflammatory response against SARS-CoV-2 leads to irreparable tissue damage. Treg responses may play a role in the intensity of response as related to COVID-19 pathogenesis. Downregulation of immune responses by regulatory T cells has been implicated in the control of pathogenic immune response in some severe diseases. Alternatively, Yang Liu et al. suggested that more severe COVID-19 disease in older human hosts may reflect more intense immune responses due to immunological memory of prior coronavirus infection, particularly with endemic human coronaviruses (57). The opposite may also be true, however, as younger individuals may have more active memory to recent coronavirus infections and more able to contain infection. It is likely that the immune response to SARS-CoV-2 is extremely complex and further studies of Treg responses in COVID-19 will provide additional insight.

## 5 Conclusion

Tregitopes were first discovered in IgG and were shown to suppress inflammatory responses to co-administered antigens both *in vitro* and *in vivo*. The co-delivery of disease-specific antigens with Tregitopes is critical to induce antigen-specific tolerance (38), which may be why human-like Tregitopes are found in antigens that are relevant to pathogen survival in the host (7). We identified an unusually human-like Treg epitope in SARS-CoV-2 that is highly conserved in the pathogen’s sequence, despite the emergence of multiple variants of concern over time. While other Treg epitopes may be present in other proteins of SARS-CoV-2, this particular Treg epitopes in SARS-CoV-2 had a surprising homology with a known Treg epitope, and we were able to confirm its tolerogenic impact *in vitro*. While future studies will define whether the same T cell clones are able to respond to the SARS epitope and the IgG Tregitope, other studies have shown that Treg and T effector T cells may recognize the same T cell epitope with different TCR specificities. Additional investigation of T cell responses to NSP7-289 will clarify the impact of this sequence and may also provide clues to SARS-CoV-2 tenacity and viral fitness.

## Data availability statement

Raw data for the figures is available upon reasonable request to EpiVax, Inc. (info@EpiVax.com).

## Ethics statement

The studies involving humans were approved by 1. Saint Louis University and 2. Sanguine Biosciences (via Advarra and WCG IRB). The studies were conducted in accordance with the local legislation and institutional requirements. The participants provided their written informed consent to participate in this study.

## Author contributions

SM: Conceptualization, Formal Analysis, Methodology, Supervision, Writing – original draft, Writing – review & editing, Data curation, Investigation. SL: Data curation, Formal Analysis, Investigation, Methodology, Supervision, Writing – original draft, Writing – review & editing. AG: Data curation, Formal Analysis, Investigation, Methodology, Writing – original draft, Writing – review & editing, Software. MM: Data curation, Formal Analysis, Investigation, Writing – review & editing. CB: Formal Analysis, Investigation, Writing – review & editing, Methodology, Supervision. LM: Formal Analysis, Investigation, Methodology, Supervision, Writing – review & editing, Conceptualization, Writing – original draft. AD: Conceptualization, Formal Analysis, Methodology, Supervision, Writing – original draft, Writing – review & editing, Project administration, Resources.

## References

[1] Worldometer.info. Worldometer COVID-19 data (2023). Available at: https://www.worldometers.info/coronavirus/.

[2] Rydyznski Moderbacher C Ramirez SI Dan JM Grifoni A Hastie KM Weiskopf D. Antigen-specific adaptive immunity to SARS-coV-2 in acute COVID-19 and associations with age and disease severity. Cell (2020) 183:996–1012.e19. doi: 10.1016/j.cell.2020.09.038 33010815 PMC7494270

[3] Zhao J Zhao J Mangalam AK Channappanavar R Fett C Meyerholz DK. Airway memory CD4 + T cells mediate protective immunity against emerging respiratory coronaviruses. Immunity (2016) 44:1379–91. doi: 10.1016/j.immuni.2016.05.006 PMC4917442 27287409

[4] De Groot AS De Groot P He Y. ICoVax 2013: the 3rd ISV pre-conference computational vaccinology workshop. BMC Bioinf (2014) 15:I1. doi: 10.1186/1471-2105-15-S4-I1 PMC4094997 25104130

[5] He L De Groot AS Gutierrez AH Martin WD Moise L Bailey-Kellogg C. Integrated assessment of predicted MHC binding and cross-conservation with self reveals patterns of viral camouflage. BMC Bioinf (2014) 15 Suppl 4:S1. doi: 10.1186/1471-2105-15-S4-S1 PMC4094998 25104221

[6] He L De Groot AS Bailey-Kellogg C. Hit-and-run, hit-and-stay, and commensal bacteria present different peptide content when viewed from the perspective of the T cell. Vaccine (2015) 33:6922–9. doi: 10.1016/j.vaccine.2015.08.099 26428457

[7] De Groot AS Moise L Liu R Gutierrez AH Tassone R Bailey-Kellogg C. Immune camouflage: Relevance to vaccines and human immunology. Hum Vaccin Immunother (2014) 10:3570–5. doi: 10.4161/hv.36134 PMC4514035 25483703

[8] Khan S Parrillo M Gutierrez AH Terry FE Moise L Martin WD. Immune escape and immune camouflage may reduce the efficacy of RTS,S vaccine in Malawi. Hum Vaccin Immunother (2020) 16:214–27. doi: 10.1080/21645515.2018.1560772 PMC7062414 30614773

[9] Liu R Moise L Tassone R Gutierrez AH Terry FE Sangare K. H7N9 T-cell epitopes that mimic human sequences are less immunogenic and may induce Treg-mediated tolerance. Hum Vaccin Immunother (2015) 11:2241–52. doi: 10.1080/21645515.2015.1052197 PMC4635734 26090577

[10] Moten D Teneva I Apostolova D Batsalova T Dzhambazov B. Molecular mimicry of the rheumatoid arthritis-related immunodominant T-cell epitope within type II collagen (CII260-270) by the bacterial L-asparaginase. Int J Mol Sci (2022) 23:9149. doi: 10.3390/ijms23169149 36012429 PMC9408948

[11] Kanjana K Strle K Lochhead RB Pianta A Mateyka LM Wang Q. Autoimmunity to synovial extracellular matrix proteins in patients with post-infectious lyme arthritis. J Clin Invest (2023) 133(17):e161170. doi: 10.1172/JCI161170 37471146 PMC10471169

[12] De Groot AS Rosenberg AS Miah SMS Skowron G Roberts BJ Lélias S. Identification of a potent regulatory T cell epitope in factor V that modulates CD4+ and CD8+ memory T cell responses. Clin Immunol (2021) 224:108661. doi: 10.1016/j.clim.2020.108661 33412295

[13] De Groot AS Moise L McMurry JA Wambre E Van Overtvelt L Moingeon P. Activation of natural regulatory T cells by IgG Fc–derived peptide “Tregitopes” Blood (2008) 112:3303–11. doi: 10.1182/blood-2008-02-138073 PMC2569179 18660382

[14] De Groot AS Moise L Terry F Gutierrez AH Hindocha P Richard G. Better epitope discovery, precision immune engineering, and accelerated vaccine design using immunoinformatics tools. Front Immunol (2020) 11:442. doi: 10.3389/fimmu.2020.00442 32318055 PMC7154102

[15] Moise L Gutierrez AH Bailey-Kellogg C Terry F Leng Q Abdel Hady KM. The two-faced T cell epitope. Hum Vaccin Immunother (2013) 9:1577–86. doi: 10.4161/hv.24615 PMC3974887 23584251

[16] Petrova G Ferrante A Gorski J. Cross-reactivity of T cells and its role in the immune system. Crit Rev Immunol (2012) 32:349–72. doi: 10.1615/CritRevImmunol.v32.i4.50 PMC3595599 23237510

[17] Cousens LP Su Y McClaine E Li X Terry F Smith R. Application of igG-derived natural treg epitopes (IgG tregitopes) to antigen-specific tolerance induction in a murine model of type 1 diabetes. J Diabetes Res (2013) 2013:1–17. doi: 10.1155/2013/621693 PMC3655598 23710469

[18] Dembele M Tao S Massoud A Zismanov V Kaufman G Lelias S. Tregitopes improve murine asthma by promoting highly suppressive and antigen specific Tregs. J Allergy Clin Immunol (2020) 145:AB79. doi: 10.1016/j.jaci.2019.12.673 PMC8089381 33953711

[19] Cousens LP Tassone R Mazer BD Ramachandiran V Scott DW De Groot AS. Tregitope update: Mechanism of action parallels IVIg. Autoimmun Rev (2013) 12:436–43. doi: 10.1016/j.autrev.2012.08.017 22944299

[20] Southwood S Sidney J Kondo A del Guercio MF Appella E Hoffman S. Several common HLA-DR types share largely overlapping peptide binding repertoires. J Immunol (1998) 160:3363–73. doi: 10.4049/jimmunol.160.7.3363 9531296

[21] Steere AC Klitz W Drouin EE Falk BA Kwok WW Nepom GT. Antibiotic-refractory Lyme arthritis is associated with HLA-DR molecules that bind a Borrelia burgdorferi peptide. J Exp Med (2006) 203:961–71. doi: 10.1084/jem.20052471 PMC3212725 16585267

[22] Greenbaum J Sidney J Chung J Brander C Peters B Sette A. Functional classification of class II human leukocyte antigen (HLA) molecules reveals seven different supertypes and a surprising degree of repertoire sharing across supertypes. Immunogenetics (2011) 63:325–35. doi: 10.1007/s00251-011-0513-0 PMC3626422 21305276

[23] Maiers M Gragert L Klitz W. High-resolution HLA alleles and haplotypes in the United States population. Hum Immunol (2007) 68:779–88. doi: 10.1016/j.humimm.2007.04.005 17869653

[24] Yin W Mao C Luan X Shen D-D Shen Q Su H. Structural basis for inhibition of the RNA-dependent RNA polymerase from SARS-CoV-2 by remdesivir. Science (80-) (2020) 368:1499–504. doi: 10.1126/science.abc1560 PMC7199908 32358203

[25] Watkin LB Mishra R Gil A Aslan N Ghersi D Luzuriaga K. Unique influenza A cross-reactive memory CD8 T-cell receptor repertoire has a potential to protect against EBV seroconversion. J Allergy Clin Immunol (2017) 140:1206–10. doi: 10.1016/j.jaci.2017.05.037 PMC5669360 28629751

[26] Meyers LM Gutiérrez AH Boyle CM Terry F McGonnigal BG Salazar A. Highly conserved, non-human-like, and cross-reactive SARS-CoV-2 T cell epitopes for COVID-19 vaccine design and validation. NPJ Vaccines (2021) 6:71. doi: 10.1038/s41541-021-00331-6 33986292 PMC8119491

[27] Reshamwala SM Likhite V Degani MS Deb SS Noronha SB. Mutations in SARS-CoV-2 nsp7 and nsp8 proteins and their predicted impact on replication/transcription complex structure. J Med Virol (2021) 93:4616–9. doi: 10.1002/jmv.26791 PMC8012999 33433004

[28] Saito R Smoot ME Ono K Ruscheinski J Wang P-L Lotia S. A travel guide to Cytoscape plugins. Nat Methods (2012) 9:1069–76. doi: 10.1038/nmeth.2212 PMC3649846 23132118

[29] Lehmann AA Reche PA Zhang T Suwansaard M Lehmann PV. CERI, CEFX, and CPI: largely improved positive controls for testing antigen-specific T cell function in PBMC compared to CEF. Cells (2021) 10:248. doi: 10.3390/cells10020248 33514016 PMC7911306

[30] Jang H Meyers LM Boyle C De Groot AS Moise L Ross TM. Immune-engineered H7N9 influenza hemagglutinin improves protection against viral influenza virus challenge. Hum Vaccin Immunother (2020) 16:2042–50. doi: 10.1080/21645515.2020.1793711 PMC7553694 32783766

[31] Urowitz MB Isenberg DA Wallace DJ. Safety and efficacy of hCDR1 (Edratide) in patients with active systemic lupus erythematosus: results of phase II study. Lupus Sci Med (2015) 2:e000104. doi: 10.1136/lupus-2015-000104 26301100 PMC4538379

[32] van Eden W Jansen MAA Ludwig IS Leufkens P van der Goes MC van Laar JM. Heat shock proteins can be surrogate autoantigens for induction of antigen specific therapeutic tolerance in rheumatoid arthritis. Front Immunol (2019) 10:279. doi: 10.3389/fimmu.2019.00279 30873163 PMC6401592

[33] Stephens LA Gray D Anderton SM. CD4 + CD25 + regulatory T cells limit the risk of autoimmune disease arising from T cell receptor crossreactivity. Proc Natl Acad Sci (2005) 102:17418–23. doi: 10.1073/pnas.0507454102 PMC1297676 16287973

[34] Weissler KA Caton AJ. The role of T-cell receptor recognition of peptide:MHC complexes in the formation and activity of Foxp3 + regulatory T cells. Immunol Rev (2014) 259:11–22. doi: 10.1111/imr.12177 24712456 PMC4034456

[35] De Groot AS Skowron G White JR Boyle C Richard G Serreze D. Therapeutic administration of Tregitope-Human Albumin Fusion with Insulin Peptides to promote Antigen-Specific Adaptive Tolerance Induction. Sci Rep (2019) 9:16103. doi: 10.1038/s41598-019-52331-1 31695065 PMC6834854

[36] van der Marel S. Adeno-associated virus mediated delivery of Tregitope 167 ameliorates experimental colitis. World J Gastroenterol (2012) 18:4288. doi: 10.3748/wjg.v18.i32.4288 22969191 PMC3436043

[37] Elyaman W Khoury SJ Scott DW De Groot AS. Potential application of tregitopes as immunomodulating agents in multiple sclerosis. Neurol Res Int (2011) 2011:1–6. doi: 10.1155/2011/256460 PMC3175387 21941651

[38] Hui DJ Basner-Tschakarjan E Chen Y Davidson RJ Buchlis G Yazicioglu M. Modulation of CD8+ T cell responses to AAV vectors with IgG-derived MHC class II epitopes. Mol Ther (2013) 21:1727–37. doi: 10.1038/mt.2013.166 PMC3776637 23857231

[39] Prangtaworn P Chaisri U Seesuay W Mahasongkram K Onlamoon N Reamtong O. Tregitope-linked refined allergen vaccines for immunotherapy in cockroach allergy. Sci Rep (2018) 8:15480. doi: 10.1038/s41598-018-33680-9 30341299 PMC6195530

[40] Cousens LP Najafian N Mingozzi F Elyaman W Mazer B Moise L. *In vitro* and *in vivo* studies of igG-derived treg epitopes (Tregitopes): A promising new tool for tolerance induction and treatment of autoimmunity. J Clin Immunol (2013) 33:43–9. doi: 10.1007/s10875-012-9762-4 PMC3538121 22941509

[41] Sela U Sharabi A Dayan M Hershkoviz R Mozes E. The role of dendritic cells in the mechanism of action of a peptide that ameliorates lupus in murine models. Immunology (2009) 128:e395–405. doi: 10.1111/j.1365-2567.2008.02988.x PMC2753948 19040426

[42] Mikecz K Glant TT Markovics A Rosenthal KS Kurko J Carambula RE. An epitope-specific DerG-PG70 LEAPS vaccine modulates T cell responses and suppresses arthritis progression in two related murine models of rheumatoid arthritis. Vaccine (2017) 35:4048–56. doi: 10.1016/j.vaccine.2017.05.009 PMC5568759 28583308

[43] Kirchdoerfer RN Ward AB. Structure of the SARS-CoV nsp12 polymerase bound to nsp7 and nsp8 co-factors. Nat Commun (2019) 10:2342. doi: 10.1038/s41467-019-10280-3 31138817 PMC6538669

[44] Gao Y Yan L Huang Y Liu F Zhao Y Cao L. Structure of the RNA-dependent RNA polymerase from COVID-19 virus. Science (80-) (2020) 368:779–82. doi: 10.1126/science.abb7498 PMC7164392 32277040

[45] Wilamowski M Hammel M Leite W Zhang Q Kim Y Weiss KL. Transient and stabilized complexes of Nsp7, Nsp8, and Nsp12 in SARS-CoV-2 replication. Biophys J (2021) 120:3152–65. doi: 10.1016/j.bpj.2021.06.006 PMC8238635 34197805

[46] Kokic G Hillen HS Tegunov D Dienemann C Seitz F Schmitzova J. Mechanism of SARS-CoV-2 polymerase stalling by remdesivir. Nat Commun (2021) 12:279. doi: 10.1038/s41467-020-20542-0 33436624 PMC7804290

[47] Pachetti M Marini B Benedetti F Giudici F Mauro E Storici P. Emerging SARS-CoV-2 mutation hot spots include a novel RNA-dependent-RNA polymerase variant. J Transl Med (2020) 18:179. doi: 10.1186/s12967-020-02344-6 32321524 PMC7174922

[48] Cobey S. Pathogen evolution and the immunological niche. Ann N Y Acad Sci (2014) 1320:1–15. doi: 10.1111/nyas.12493 25040161 PMC4141700

[49] Bedoya F Cheng G-S Leibow A Zakhary N Weissler K Garcia V. Viral antigen induces differentiation of foxp3+ Natural regulatory T cells in influenza virus–infected mice. J Immunol (2013) 190:6115–25. doi: 10.4049/jimmunol.1203302 PMC3703618 23667113

[50] Kurup SP Obeng-Adjei N Anthony SM Traore B Doumbo OK Butler NS. Regulatory T cells impede acute and long-term immunity to blood-stage malaria through CTLA-4. Nat Med (2017) 23:1220–5. doi: 10.1038/nm.4395 PMC5649372 28892065

[51] Bunn PT Montes de Oca M de Labastida Rivera F Kumar R Ng SS Edwards CL. Distinct Roles for CD4+ Foxp3+ Regulatory T Cells and IL-10–Mediated Immunoregulatory Mechanisms during Experimental Visceral Leishmaniasis Caused by Leishmania donovani. J Immunol (2018) 201:3362–72. doi: 10.4049/jimmunol.1701582 30355785

[52] Trandem K Anghelina D Zhao J Perlman S. Regulatory T cells inhibit T cell proliferation and decrease demyelination in mice chronically infected with a coronavirus. J Immunol (2010) 184:4391–400. doi: 10.4049/jimmunol.0903918 PMC2851486 20208000

[53] Martins R Carlos AR Braza F Thompson JA Bastos-Amador P Ramos S. Disease tolerance as an inherent component of immunity. Annu Rev Immunol (2019) 37:405–37. doi: 10.1146/annurev-immunol-042718-041739 30673535

[54] Neumann J Prezzemolo T Vanderbeke L Roca CP Gerbaux M Janssens S. Increased IL-10-producing regulatory T cells are characteristic of severe cases of COVID-19. Clin Transl Immunol (2020) 9:e1204. doi: 10.1002/cti2.1204 PMC7662088 33209300

[55] Cantor H. T-cell receptor crossreactivity and autoimmune disease. Advances in Immunology (2000) 75:209–33. doi: 10.1016/S0065-2776(00)75005-X 10879285

[56] Casadevall A Pirofski L. In fatal COVID-19, the immune response can control the virus but kill the patient. Proc Natl Acad Sci (2020) 117:30009–11. doi: 10.1073/pnas.2021128117 PMC7720099 33177233

[57] Liu Y Mao B Liang S Yang J-W Lu H-W Chai Y-H. Association between age and clinical characteristics and outcomes of COVID-19. Eur Respir J (2020) 55:2001112. doi: 10.1183/13993003.01112-2020 32312864 PMC7173682

