# Supplementary material for: A SARS-CoV-2 NSP7 homolog of a Treg epitope suppresses CD4+ and CD8+ T cell memory responses
Source: Front Immunol. 2023 Nov 24;14:1290688. doi: 10.3389/fimmu.2023.1290688 (PMC10731459; doi:10.3389/fimmu.2023.1290688)
Supplement: Supplementary file 1 [file DataSheet_1.pdf]

Supplemental Data for:

A SARS-CoV-2 NSP7 homolog of a Treg epitope suppresses CD4+ and CD8+ T cell memory responses

Shah Md. Shahjahan Miah , Sandra Lelias , Andres H. Gutierrez, Mitchell McAllister, Christine M. Boyle, Lenny Moise, Anne S. De Groot

Supplementary Table 1. Healthy donors used in Bystander Supression Assays (BSA).

Donor	Allele 1	Allele 2	Gender	Age	Donor	Allele 1	Allele 2	Gender	Age
EV202	*0301	*1501	Female	36	EV327	*0405	*1401	Male	54
EV229	*0101	*0301	Male	20	EV329	*1201	*1501	Female	49
EV277	*1401	*1501	Male	54	EV354	*0101	*1301	Male	48
EV279	*0701	*1101	Male	71	EV366	*0406	*1601	Female	54
EV285	*0101	*1302	Female	54	EV375	*0301	*1501	Female	46
EV292	*0301	*1301	Male	57	EV381	*0301	*1303	Female	57
EV299	*0301	*0701	Male	67	EV545	*0401	*0701	Male	40
EV318	*0402	*0701	Male	69	EV546	*1101	*1501	Female	34
EV320	*0701	*0701	Male	51					

Supplementary Table 1. Healthy blood donors collected from Rhode Island Blood Center from whom we have isolated PBMCs and stored at liquid N2 before use. The effect of Tregitope peptides and newly identified SARS-CoV-2-NSP7-homolog of 289 Tregitope were tested using our in vitro bystander suppression assays. HLA DRB1 of donor PBMCs were typed at American Red Cross in Dedham, MA. **41% percent of the donors were female, 59% were male. The age range of donors are 20 to 69.**

Supplementary Table 2. Convalescent COVID-19 donors used in CPI BSA.

Donor	Allele 1	Allele 2	Gender	Age
CV001	*0701	*1101	Female	56
CV003	*1502	*1502	Female	57
CV020	*1301	*1501	Male	40
L009	*0401	*1201	Female	54
L011	*0301	*1101	Female	28
L013	*0801	*1303	Female	43
L014	*0101	*1401	Male	37

Supplementary Table 2. List of the convalescent COVID-19 blood donors whom we have isolated PBMCs and stored at liquid N2 before use. The effect of Tregitope peptides and newly identified SARS-CoV2-NSP7-homolog of 289 Tregitope were tested using our in vitro CPI bystander supression assay. HLA DRB1 of donor PBMCs were typed at American Red Cross in Dedham, MA. **72% percent of the donors were female, 28% were male. The age range of donors are 28 to 57.**
CPI = a mixture of protein antigens from the cytomegalovirus, influenza, and parainfluenza.

Supplement Figure S1. Overview of Class II JanusMatrix Results of the SARS-CoV-2 NSP7- homolog of Tregitope 289 peptide.

Input Sequence	Cluster Address (w/ Flanks)	Cluster Sequence	EpiMatrix Cluster Score (w/o Flanks)	Treg-Adj Cluster Score (w/o Flanks)	Number of HUMAN Matches	Janus Homology Score
NSP7_289HOMOLOG	50 - 62	**EKMVSLLSVLLSM**	31.97	31.97	74	11.44

Protein ID	Protein Description	Start Position	Sequence	Cluster Score	Number Of HUMAN Matches*	Janus HMLGY Score	DRB1 *0101	DRB1 *0301	DRB1 *0401	DRB1 *0701	DRB1 *0801	DRB1 *0901	DRB1 *1101	DRB1 *1301	DRB1 *1501
NSP7_289HOMOLOG		50 - 62	EKMVSLLSVLLSM	31.97	73	11.44	DB Ver: April 02, 2023			EpiMatrix Ver: 1.2 with DR9			JMX Hit Threshold: 1.64		
		50	EKMVSLLSV		0		0.23	0.48	0.19	0.99	0.13	1.09	1.1	0.83	1.27
		51	KMVSLLSVL		0		-0.08	0.94	-0.51	0.73	-0.3	1.15	0.09	0.49	0.68
		52	MVSLLSVLL		20	F52	3.12	2.25	2.42	2.97	2.18	2.08	1.74	2.43	2.84
sp|Q9C0C7|AMRA1_HUMAN	Activating molecule in BECN1-regula...	444	SVSLLSVLR				1.79	1.61	1.48	1.17	1.67	0.83	0.74	1.8	1.49
sp|O00206|TLR4_HUMAN	Toll-like receptor 4	635	GVSVLSVLV				1.55	0.87	1.15	1.99	0.32	1.52	0.51	1.06	1.48
sp|Q13873|BMPR2_HUMAN	Bone morphogenetic protein receptor...	157	SVSVLAVLI				1.71	1.57	0.91	1.8	0.71	1.77	0.41	1.74	1.57
sp|P01861|IGHG4_HUMAN	Immunoglobulin heavy constant gamma...	182	VVSVLTVLH				1.68	1.85	2.42	1.38	2.1	1.9	1.67	2.02	1.52
sp|P01860|IGHG3_HUMAN	Immunoglobulin heavy constant gamma...	232	VVSVLTVLH				1.68	1.85	2.42	1.38	2.1	1.9	1.67	2.02	1.52
sp|P01857|IGHG1_HUMAN	Immunoglobulin heavy constant gamma...	185	VVSVLTVLH				1.68	1.85	2.42	1.38	2.1	1.9	1.67	2.02	1.52
sp|Q8NGI9|OR5A2_HUMAN	Olfactory receptor 5A2	209	IVSVLVVLI				2.27	2.35	1.81	2.57	1.56	2.57	1.25	2.51	1.57
sp|Q5XPI4|RN123_HUMAN	E3 ubiquitin-protein ligase RNF123	1078	SVSLLRVLE				0.65	1.42	-0.14	0.16	2.04	-0.22	0.58	1.61	0.4
sp|P78395|PRAME_HUMAN	Melanoma antigen preferentially exp...	347	SVSQLSVLS				1.9	1.59	2.04	0.85	1.42	0.46	1.29	2.27	1.69
sp|Q05707|COEA1_HUMAN	Collagen alpha-1(XIV) chain	690	EVSLLAVLD				1.42	0.23	0.89	0.98	1.92	0.55	0.27	0.45	0.52
sp|P49796|RGS3_HUMAN	Regulator of G-protein signaling 3	81	HVSVLSVLS				1.86	1.31	1.92	1.45	1.21	1.59	1.32	1.49	1.64
sp|Q9BXR5|TLR10_HUMAN	Toll-like receptor 10	70	SVSKLRVLI				0.62	1.76	-0.86	0.98	1.63	0.53	0.33	2.17	0.37
sp|Q8NCM8|DYHC2_HUMAN	Cytoplasmic dynein 2 heavy chain 1	3785	VVSWLPVLE				0.47	1.25	1.45	1	1.29	0.83	0.27	2.21	0.61
sp|Q5TH69|BIG3_HUMAN	Brefeldin A-inhibited guanine nucle...	206	VVSVLTVLC				2	1.24	1.81	1.77	1.42	1.73	1.03	1.42	1.84
sp|O75746|S2512_HUMAN	Electrogenic aspartate/glutamate an...	465	RVSALNVLR				1.26	2.34	1.33	0.66	1.36	1.58	0.09	2.28	1.69
sp|Q9HB89|NMUR1_HUMAN	Neuromedin-U receptor 1	103	AVSDLLVLL				0.12	1.83	0.42	-0.16	0.35	0.27	-0.66	0.71	0.3
sp|Q9GZQ4|NMUR2_HUMAN	Neuromedin-U receptor 2	88	AVSDLLVLL				0.12	1.83	0.42	-0.16	0.35	0.27	-0.66	0.71	0.3
sp|Q6NW34|NEPRO_HUMAN	Nucleolus and neural progenitor pro...	179	LVSRLWVLY				0.82	1	0.2	0.88	0.45	0.58	-0.04	2.03	1.25
sp|Q9Y5I1|PCDAB_HUMAN	Protocadherin alpha-11	705	VVSSLLVLT				1.25	1.65	1.02	1.55	1.2	2.2	1.27	1.28	1.89
sp|Q8NCA5|FA98A_HUMAN	Protein FAM98A	49	LVSELRVLC				1.99	1.63	0.86	1.24	1.13	0.9	1	1.38	0.92
		53	VSLLSVLLS		36	F53	2.09	1.68	2.59	1.44	1.89	1.25	2.47	2.03	1.58
sp|P30408|T4S1_HUMAN	Transmembrane 4 L6 family member 1	160	VSLFSILLA				0.38	0.47	1.57	1.1	0.85	1.32	1.44	1.83	1.88
sp|Q5VT06|CE350_HUMAN	Centrosome-associated protein 350	756	GSLLSHLLS				0.8	0.68	1.21	0.16	1.07	0.04	1.68	1.04	1.49
sp|Q9NX18|SDHF2_HUMAN	Succinate dehydrogenase assembly fa...	19	HSLLSPLLS				1.7	0.8	1.84	0.91	1.33	0.89	1.93	1.16	1.29
sp|Q5JRX3|PREP_HUMAN	Presequence protease, mitochondrial	346	LSLLSSLLT				2.66	0.97	2.57	2.61	1.89	1.84	2.4	1.32	2.8
sp|Q76KD6|SPERI_HUMAN	Speriolin	193	VSLSSPLLS				1.97	2.4	2.2	1.78	1.31	2.31	2.23	2.17	2.04
sp|P13866|SC5A1_HUMAN	Sodium/glucose cotransporter 1	145	LSLLSLLLY				0.93	1.99	0.95	0.44	0.99	1.22	1.78	2.33	1.88
sp|Q96M86|DNHD1_HUMAN	Dynein heavy chain domain-containin...	1568	TSLLSALLV				1.76	0.38	1.46	1.74	0.71	0.94	1.38	0.75	1.34
sp|Q9Y4D7|PLXD1_HUMAN	Plexin-D1	1410	ISLFSSLLN				1.68	0.39	2.22	1.71	1.86	1.73	1.74	1.75	2.89
sp|Q9GZU5|NYX_HUMAN	Nyctalopin	414	SSLLSKLLA				0.71	1.18	0.66	-0.1	1.2	0.71	1.96	1.54	1.42
sp|P59826|BPIB3_HUMAN	BPI fold-containing family B member...	167	ISLFSGLLP				1.07	0.06	1.78	0.83	0.88	1.68	1.22	1.43	1.85
sp|P40197|GPV_HUMAN	Platelet glycoprotein V	207	VSLDSGLLN				1.33	1.35	1.89	0.4	0.79	0.84	0.24	0.39	0.91
sp|A0A1B0GUA7|TEX51_HUMAN	Testis-expressed protein 51	142	VSLSSALLL				2.3	2.4	1.89	2.81	1.07	2.45	1.75	2.18	2.44
sp|P19021|AMD_HUMAN	Peptidyl-glycine alpha-amidating mo...	391	YSLLSKLLG				2.12	2.03	1.95	0.63	3.26	1.7	3.32	2.37	2.28
sp|Q9C0I3|CCSE1_HUMAN	Serine-rich coiled-coil domain-cont...	240	SSLQSPLLS				1.25	1.1	1.55	0.32	0.92	0.14	1.58	1.95	1.27
sp|Q96RG2|PASK_HUMAN	PAS domain-containing serine/threon...	1225	MSLVSGLLQ				1.63	1.03	1.96	1.62	0.96	1.26	1.81	1.37	1.88
sp|O14497|ARI1A_HUMAN	AT-rich interactive domain-containi...	1674	MSLKSGLLA				1.1	1.12	1.02	1.05	1.15	0.91	1.22	1.7	0.97
sp|Q6ZP01|RBM44_HUMAN	RNA-binding protein 44	683	LSLESKLLS				1.49	2.42	1.72	0.28	1.3	1.19	2.44	2.31	1.4

Protein ID	Protein Description	Start Position	Sequence	Cluster Score	Number Of HUMAN Matches*	Janus HMLGY Score	DRB1 *0101	DRB1 *0301	DRB1 *0401	DRB1 *0701	DRB1 *0801	DRB1 *0901	DRB1 *1101	DRB1 *1301	DRB1 *1501
sp|Q9Y243|AKT3_HUMAN	RAC-gamma serine/threonine-protein ...	374	KSLLSGLLI				1.95	0.75	1.19	1.51	0.7	1.02	0.89	1.11	1.55
sp|Q8NFD5|ARI1B_HUMAN	AT-rich interactive domain-containi...	1711	MSLKSGLLA				1.1	1.12	1.02	1.05	1.15	0.91	1.22	1.7	0.97
sp|Q8N755|S66A3_HUMAN	Solute carrier family 66 member 3	37	LSLPSLLLE				-0.95	1.1	-0.09	-0.45	1.02	-0.42	0.16	1.89	0.74
sp|Q6ZS81|WDFY4_HUMAN	WD repeat- and FYVE domain-containi...	1481	LSLFSHLLE				0.12	0.95	1.01	0.33	2.03	0.46	1.18	2.3	1.99
sp|Q5TEC3|ZN697_HUMAN	Zinc finger protein 697	326	FSLSSHLLS				1.93	2.7	2.4	1.88	1.95	2.33	2.86	2.47	2.64
sp|Q8TBY8|PMFBP_HUMAN	Polyamine-modulated factor 1-bindin...	13	SSLNSKLLS				0.51	1.69	0.71	-0.51	1.4	0.28	2.01	1.82	1.2
sp|P55290|CAD13_HUMAN	Cadherin-13	697	FSLPSVLLL				1.51	1.51	1.89	2.05	1.04	1.33	1.19	2.29	1.41
sp|Q4KMG0|CDON_HUMAN	Cell adhesion molecule-related/down...	85	LSLNSSLLG				2.18	2.06	2.55	1.43	2.27	1.18	2.38	2.18	2.31
sp|A6ND91|ASPDH_HUMAN	Aspartate dehydrogenase domain-cont...	19	QSLVSRLLA				0.73	0.74	0.31	0.74	0.62	0.89	1.68	1.08	0.89
sp|P55198|AF17_HUMAN	Protein AF-17	541	LSLESPLLG				2.01	1.79	2.27	0.91	1.09	1.17	1.75	1.69	1.13
sp|Q96MX3|ZNF48_HUMAN	Zinc finger protein 48	368	FSLSSTLLR				2.04	2.8	2.49	2.07	1.9	2.29	2.06	2.57	1.85
sp|Q9BV99|LRC61_HUMAN	Leucine-rich repeat-containing prot...	28	FSLESILLL				1.73	1.77	1.99	2.62	0.62	1.62	1.54	1.67	1.13
sp|Q9NUD9|PIGV_HUMAN	GPI mannosyltransferase 2	109	LSLRSCLLI				0.78	0.68	0.82	1.26	0.99	0.67	0.65	1.88	1.1
sp|Q9UPM8|AP4E1_HUMAN	AP-4 complex subunit epsilon-1	846	FSLTSELLD				0.42	0.18	0.44	1.57	1.85	0.91	0.33	1.14	0.91
sp|Q68CQ1|MROH7_HUMAN	Maestro heat-like repeat-containing...	337	LSLDSSLLF				1.19	2.71	2.57	1.6	1.27	1.57	1.02	1.73	1.15
sp|Q8NH41|OR4KF_HUMAN	Olfactory receptor 4K15	206	LSLSSFLLL				1.18	1.88	0.94	2.29	0.6	1.58	1.3	1.66	1.44
sp|O14771|ZN213_HUMAN	Zinc finger protein 213	410	FSLRSYLLD				1.22	-0.31	-0.11	1.23	1.75	0.43	0.36	0.91	1.16
sp|O43286|B4GT5_HUMAN	Beta-1,4-galactosyltransferase 5	22	FSLSSSLLY				2.24	2.94	2.29	2.33	0.92	2.94	2.03	2.71	2.31
sp|Q8N6C8|LIRA3_HUMAN	Leukocyte immunoglobulin-like recep...	208	WSLPSDLLG				0.39	0.17	1.92	0.05	1.15	-0.06	1.06	0.98	1.11
		54	SLLSVLLSM		17	F54	0.08	1.81	0.03	0.58	-0.44	1.52	0.74	1.6	1.25
sp|Q9Y6C5|PTC2_HUMAN	Protein patched homolog 2	1105	VLLPVLLSI				0.3	1.73	0.47	0.89	0.76	1.01	0.74	2.51	2.03
sp|O60241|AGRB2_HUMAN	Adhesion G protein-coupled receptor...	19	LLLSVILSL				1.53	2.31	1.65	3.05	1.05	2.61	1.73	2.09	2.04
sp|Q9P2K9|DISP3_HUMAN	Protein dispatched homolog 3	1207	LLLPVLLSI				0.43	1.79	0.59	1.01	0.9	1.13	0.87	2.56	2.08
sp|Q86Y46|K2C73_HUMAN	Keratin, type II cytoskeletal 73	419	ELLSVKLSL				0.99	1.86	0.42	1.18	0.94	1.93	1.62	1.64	1.46
sp|O60423|AT8B3_HUMAN	Phospholipid-transporting ATPase IK	1189	ILLVVLLSV				1.28	1.89	1.21	2.03	1.24	2.19	2.18	2.21	2.61
sp|A4D0T7|SIM30_HUMAN	Small integral membrane protein 30	33	LLLGVVLSI				1.65	2.2	1.76	1.98	1.01	1.68	1.18	1.86	1.59
sp|Q8IWT1|SCN4B_HUMAN	Sodium channel subunit beta-4	23	FLLPVTLSL				2.06	2	2.57	2.38	1.55	1.76	1.69	2.78	2.4
sp|Q15166|PON3_HUMAN	Serum paraoxonase/lactonase 3	8	VLLGVGLSL				2.11	1.79	1.73	1.92	0.69	1.45	1.05	1.46	2.4
sp|Q9NTG1|PKDRE_HUMAN	Polycystin family receptor for egg ...	8	LLLGVGLSL				2.24	1.84	1.85	2.04	0.82	1.58	1.18	1.51	2.45
sp|Q9BUM1|G6PC3_HUMAN	Glucose-6-phosphatase 3	153	FLLAVGLSR				2.55	2.57	2.47	2.03	2.24	2.74	1.72	2.66	2.48
sp|Q86TG1|T150A_HUMAN	Transmembrane protein 150A	5	ILLPVSLSA				1.56	1.72	2.33	1.73	1.07	1.56	1.62	2.5	2.42
sp|P04118|COL_HUMAN	Colipase	6	ILLLVALSV				3.02	2.02	2.67	2.98	2.02	2.33	2.66	2.36	2.91
sp|H3BQJ8|LY6L_HUMAN	Lymphocyte antigen 6L	125	LLLQVGLSL				2.82	1.91	2.25	2.25	1.56	1.44	1.95	2.74	2.61
sp|P39060|COIA1_HUMAN	Collagen alpha-1(XVIII) chain	534	VLLGVKLSG				0.98	2.39	1.16	-0.27	1.99	1.07	2.09	2.04	2.21
sp|O15120|PLCB_HUMAN	1-acyl-sn-glycerol-3-phosphate acyl...	14	LLLLVQLSR				1.85	1.82	1.29	1.4	1.95	1.44	1.78	2.16	1.4
sp|Q14667|BLTP2_HUMAN	Bridge-like lipid transfer protein ...	10	VLLLVALSA				2.64	1.96	2.73	2.13	1.98	2.05	2.72	2.3	2.61
sp|O95994|AGR2_HUMAN	Anterior gradient protein 2 homolog	9	FLLLVALSY				2.9	2.65	2.7	2.12	1.92	2.58	2.69	2.97	2.57

* Count of HUMAN JanusMatrix matches found in the search database. With respect to a given EpiMatrix Hit (a 9-mer contained within the input sequence which is predicted to bind to a specific allele) , a Janus Matrix match is a 9-mer derived from the search database (typically the human genome) which is predicted to bind to the same allele as the EpiMatrix Hit and shares TCR facing contacts with the EpiMatrix Hit.

** Janus Homology Score represents the average depth of coverage in the search database for each EpiMatrix hit in the input sequence. For example, an input peptide with eight EpiMatrix hits, all of which have one match in the search database, has a Janus Homology Score of 1. An input peptide with four EpiMatrix Hits, all of which have two matches in the search database, has a Janus Homology Score of 2.

The JanusMatrix Homology Score considers all constituent 9-mers in any given peptide, including flanks.

Supplement Figure S1. JanusMatrix Analysis of the SARS-CoV-2 NSP7- homolog of Tregitope 289 peptide. The JanusMatrix algorithm evaluates predicted MHC class II T cell epitopes for their homology to the human proteome. Here we observed that the NSP7 peptide (50-62) has shared homology to some common human protein sequences with a total of 73 human matches. Three epitopes were identified in frames 52, 53 and 54 that show significant cross-conservation. As shown in the above report frame 52 has 100% homology at the TCR-face (homologous amino acids are underlined) which corresponds to the well- characterized regulatory epitope, Tregitope 289, that is present in the constant region of human immunoglobin (the 9-mer epitope is shown in the green box). Tregitope regulatory epitopes are highlighted in green in this report, and frames that predict binding across multiple alleles (≥ 4) are considered promiscuous epitopes and have a higher likelihood to impact a larger global population. Additionally, there is cross-conservation in frames 53 and 54, having 36 and 17 human matches, respectively; these epitopes may be contributing to the HLA binding and the suppressive capacity observed in our bystander suppressive T cell assays for this peptide.

Supplement Figure S2. Flow cytometry gating strategy for CD4 T cell proliferation and activation in the bystander suppression assays.

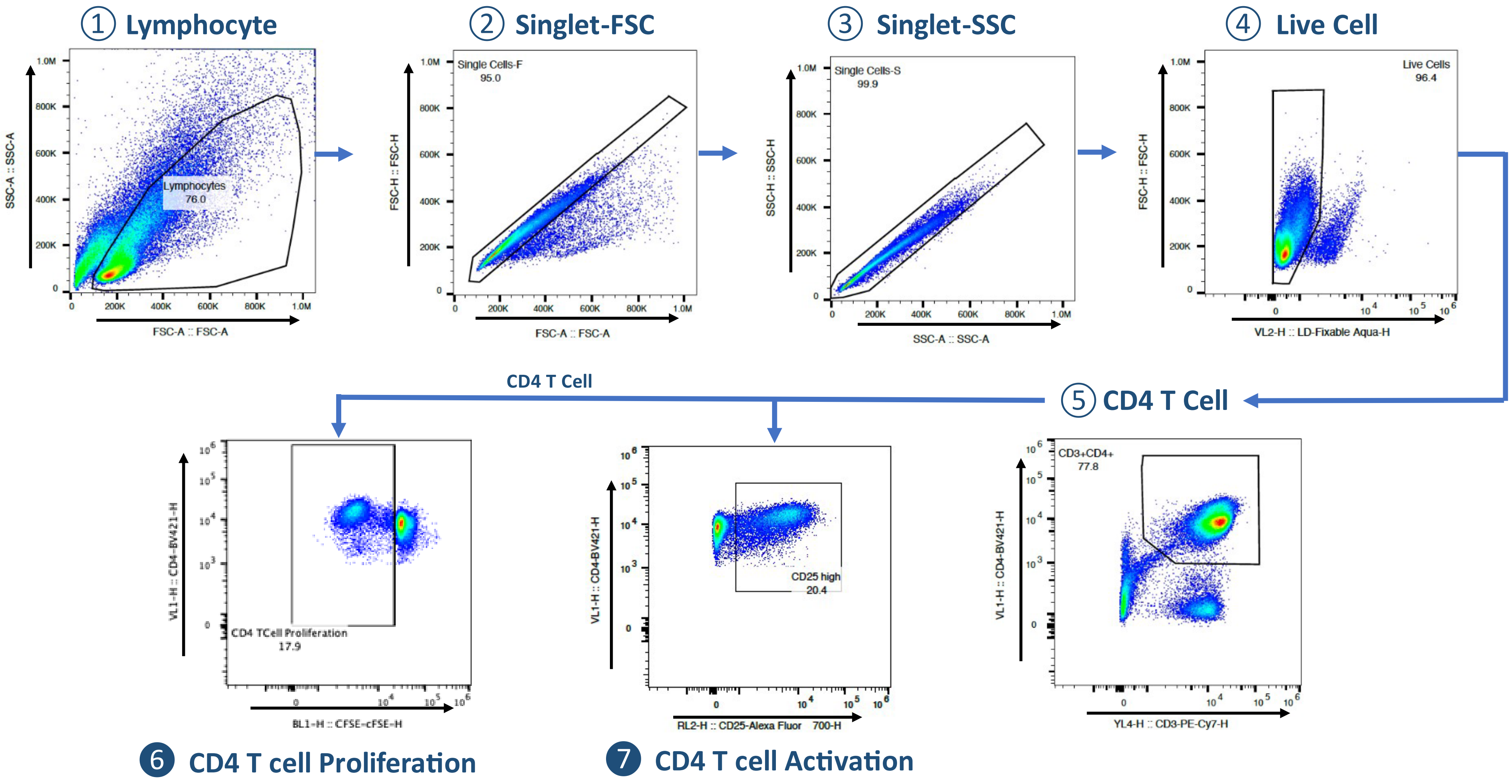

Supplement Figure S2. Gating strategy for CD4 T cell proliferation and activation. Healthy donors PBMCs were CFSE labelled at day0, then stimulated with 0.5 µg/ml of TT at day1, cultured for 6 days and stained with cell surface markers to identify CD4 T cell proliferation and activation. Initially lymphocyte populations were selected in side scatter (SSC) vs forward scatter (FSC) and doublets were excluded by flow scatter height vs area and side scatter height vs area plotting. Dead cells were then removed by live-dead staining. CD4 +T cells were selected by CD3+CD4+ positive markers and proliferation of CD4 T cells determined by CFSE dilution. Activation of CD4 T cells population was determined by the expression of CD25.

Supplement Figure S3. Representive FACS plot showing dose-dependent inhibition of CD4 T cell activation.

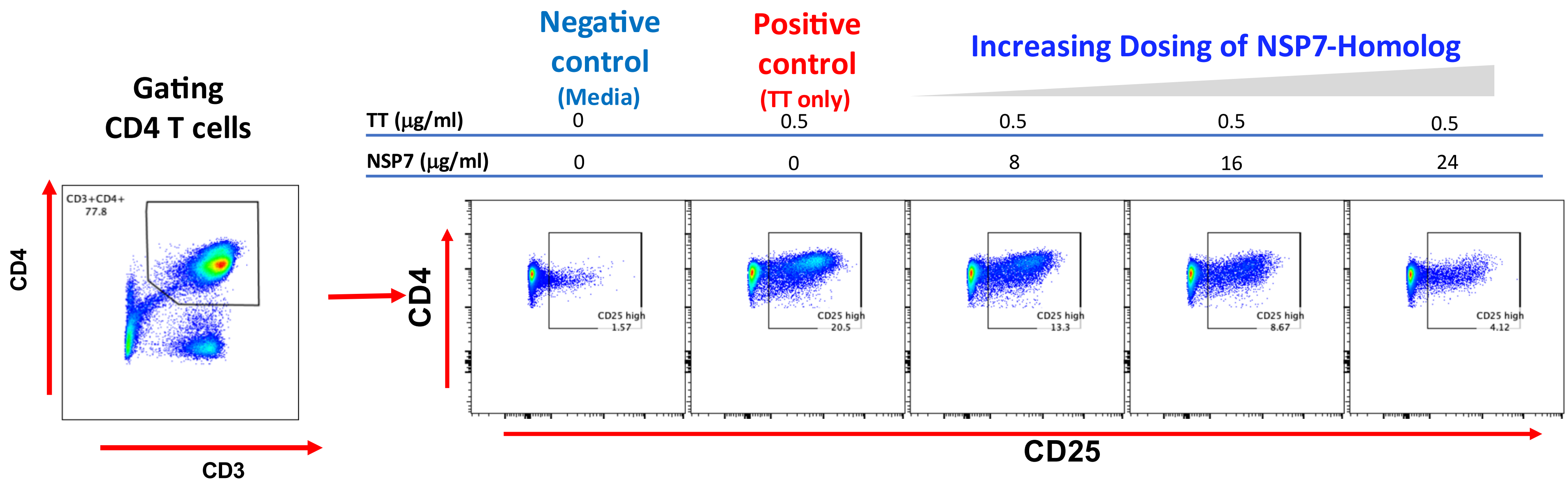

Supplement Figure S3. FACS plot showing dose-dependent inhibition of CD4 T cell activation. PBMC from healthy donors were stimulated with 0.5 ug/ml of TT with or without the addition of different concentrations of NSP7-Homolog peptide. Activation of CD4 T cells were measured six days post-stimulation by flow cytometry by the expression of activation marker CD25. FACS plot from a representative donor PBMC is shown here.
